# Antipsychotic Drugs and the Risk of Diabetic Complications: A Systematic Review of Observational Studies

**DOI:** 10.3390/jcm15093536

**Published:** 2026-05-06

**Authors:** Nisrine Haddad, Nawal Farhat, Christopher A. Gravel, Yue Chen, Franco Momoli, Donald R. Mattison, Jeannette Goguen, Daniel Krewski

**Affiliations:** 1 School of Epidemiology and Public Health, University of Ottawa, Ottawa, ON K1H 8M5, Canada; 2 School of Mathematics and Statistics, Carleton University, Ottawa, ON K1S 5B6, Canada; 3 Department of Mathematics and Statistics, University of Ottawa, Ottawa, ON K1N 6N5, Canada; 4 Data Literacy Research Institute, University of Ottawa, Ottawa, ON K1N 6N5, Canada; 5 Risk Sciences International, Ottawa, ON K1Z 7T1, Canada; 6 Arnold School of Public Health, University of South Carolina, Columbia, SC 29208, USA; 7 Department of Medicine, University of Toronto, Toronto, ON M5S 3H2, Canada; 8 Division of Endocrinology, St. Michael’s Hospital, Toronto, ON M5C 2T2, Canada

**Keywords:** antipsychotic drugs, diabetic ketoacidosis, hyperglycemic hyperosmolar state, analytical studies, descriptive studies

## Abstract

**Background:** In recent years, case reports and case series have suggested an association between the use of second- (SGAs), but not first-generation antipsychotics (FGAs), also known as atypical and typical APDs, respectively, and hyperglycemic complications, notably diabetic ketoacidosis (DKA) and hyperglycemic hyperosmolar state (HHS). Although this evidence is informative, there is a need for more observational studies to strengthen this body of knowledge. **Objective:** To conduct a systematic review of evidence established in observational studies on adverse drug events, specifically DKA and HHS, associated with the use of FGAs and SGAs. **Methods:** Pertinent bibliographic databases (MEDLINE, EMBASE, PsycINFO, and the Cochrane Central Register of Controlled Trials (CENTRAL)) were searched using appropriate index phrases and keywords through October 17, 2025. Exposure included at least one United States Food and Drugs Administration (US FDA)-approved antipsychotic drug (APD); outcomes were limited to DKA and HHS. **Results:** A total of 15 observational studies were included in this review, including seven analytical and eight descriptive studies. These studies varied in scope and used different case definitions, study populations, exposures, and outcomes. The observational studies support existing evidence of an association between atypical APDs and DKA, mainly. As a class, typical APDs were associated with an increased risk of DKA, when compared to non-antipsychotic drug use. Although some studies evaluated this association in relation to HHS, there is insufficient information to draw conclusions for this outcome at this time. **Conclusions:** This analysis provides additional evidence of an association between use of atypical APDs and DKA. Additional analytical studies using administrative health databases are needed to clarify this association.

## 1. Introduction

The prevalence of diabetes in individuals treated with antipsychotic drugs (APDs) for mental illness is about 10%, 2–3-fold higher than in the general population [1,2,3]. APDs are indicated primarily to treat schizophrenia, bipolar disorder, and other psychotic disorders [4]; however, these medicines are also used “off-label” to treat other conditions such as depression, anxiety, Tourette’s syndrome, personality disorders, and obsessive-compulsive disorders [1,5]. The underlying mechanisms of action of APDs are not fully understood, but accrued evidence suggests they play a role in the etiology of diabetes and hyperglycemic emergencies [1]. A meta-analysis comparing drug-naïve subjects presenting with a first episode of schizophrenia to control subjects found that metabolic health problems may be present at onset of the mental health condition, and that patients are at an increased risk of developing diabetes mellitus (DM) [6]. First-generation antipsychotic drugs (FGAs), also known as typical antipsychotics, are effective in treating positive symptoms of schizophrenia. However, their use is associated with significant extrapyramidal side effects, including dystonia, dyskinesia, akathisia, and parkinsonism [4,7] as well as hyperprolactinemia, primarily due to their antagonistic properties acting on dopamine receptors in the nigrostriatal and tuberoinfundibular pathways [1]. These agents exert their therapeutic effects primarily through modulation of dopamine D2 receptors in the mesolimbic and mesocortical pathways, while also exhibiting variable blockade effects on muscarinic, histaminergic and adrenergic receptors [1]. Coupled with their limited efficacy in treating negative and cognitive symptoms, such as social withdrawal and apathy [8,9,10] and poorer patient adherence to treatment [11], these concerns paved the way for the development of second-generation antipsychotics (SGAs), also known as atypical APDs. SGAs include clozapine, an effective agent in treatment-resistant schizophrenia [12,13], as well as other drugs with broader receptor profiles that are associated with improved tolerability and adherence, and a reduced manifestation of extrapyramidal adverse effects [14]. SGAs have a lower affinity for dopamine D2 receptors overall, and are also antagonists of 5-hydroxytryptamine type 2 (5HT2) serotonin receptors. Some agents, such lurasidone and aripiprazole, are strong 5HT1a agonists [15]. The latter has unique pharmacological properties and manifests a partial D2 agonist [16].

In recent years, evidence has accrued correlating the use of atypical APDs with adverse health outcomes including increased weight gain and the development of diabetes and dyslipidemia. This evidence prompted the American Diabetes Association, the American Psychiatric Association, the American Association of Clinical Endocrinologists, and the North American Association for the Study of Obesity to publish a consensus statement on the risk of these and other adverse health effects in patients treated with atypical APDs [17].

Diabetes, a metabolic disorder, is a multifactorial disease involving genetic, lifestyle, and other risk factors. As such, it is difficult to determine with certainty if the use of APDs causes diabetes or hyperglycemic complications. Investigation of this association is made more challenging due low patient compliance with treatment; it is estimated that less than 50% of schizophrenia patients are fully adherent to APD treatment [18]. Guo et al. (2023) [19] conducted a meta-analysis and reported five potential risk factors that impact medication adherence in people with schizophrenia. These include: (1) drug-related factors such as drugs taken, their therapeutic and side effects; (2) disease factors such as duration and severity of disease and concomitant physical illnesses; (3) problem behaviors such as alcohol and substance abuse; (4) low income and poor quality of life; and (5) personal characteristics including age, gender, and education. However, the authors also report that a high level of support, positive attitude, and healthy behaviors appear to be protective factors. To address this issue, new approaches were introduced to enhance compliance to the prescribed treatment plan such as utilizing new routes of administration including short-acting intramuscular clozapine preparation, transdermal asenapine, modification of existing compounds, and oral long-acting medication formulation [20].

Absolute insulin deficiency resulting from autoimmune-mediated destruction of pancreatic ß cells characterizes Type 1 diabetes mellitus (T1DM), also known as autoimmune diabetes, a chronic disease with elevated blood sugar levels [21]. In contrast, type 2 diabetes mellitus (T2DM) is characterized by insulin resistance accompanied by abnormal ß cell activity, resulting in relative insulin deficiency and decreased insulin secretion [22]. Both conditions reduce peripheral glucose uptake and increase hepatic glucose production, thereby contributing to hyperglycemia [23,24].

Diabetic ketoacidosis (DKA) and hyperosmolar hyperglycemic state (HHS) are two potentially fatal acute hyperglycemic complications of diabetes mellitus that result from severe insulin deficiency and require prompt, intensive medical management, including insulin therapy and aggressive fluid resuscitation [25,26]. Patients with type 1 diabetes mellitus (T1DM) are at higher individual risk of developing DKA, which occurs more frequently than HHS. However, epidemiological data indicate that although DKA is more common in patients with T1DM, up to one-third of DKA cases occur in patients with type 2 diabetes mellitus (T2DM), reflecting the increasing burden of DKA in this population [27].

Recent systematic reviews of case reports and case series support accumulating evidence of an association between atypical APDs and DKA [26,28,29] and HHS [26]. The objective of this review is to evaluate and synthesize information from observational studies to shed light on the associations between antipsychotic medications and hyperglycemic emergencies, specifically DKA and HHS.

## 2. Methods

This review employed the same search strategy as our previously published systematic review of case reports on DKA and HHS [26], with modifications detailed below to include observational study designs.

### 2.1. Research Strategy and Databases

**Figure 1:**
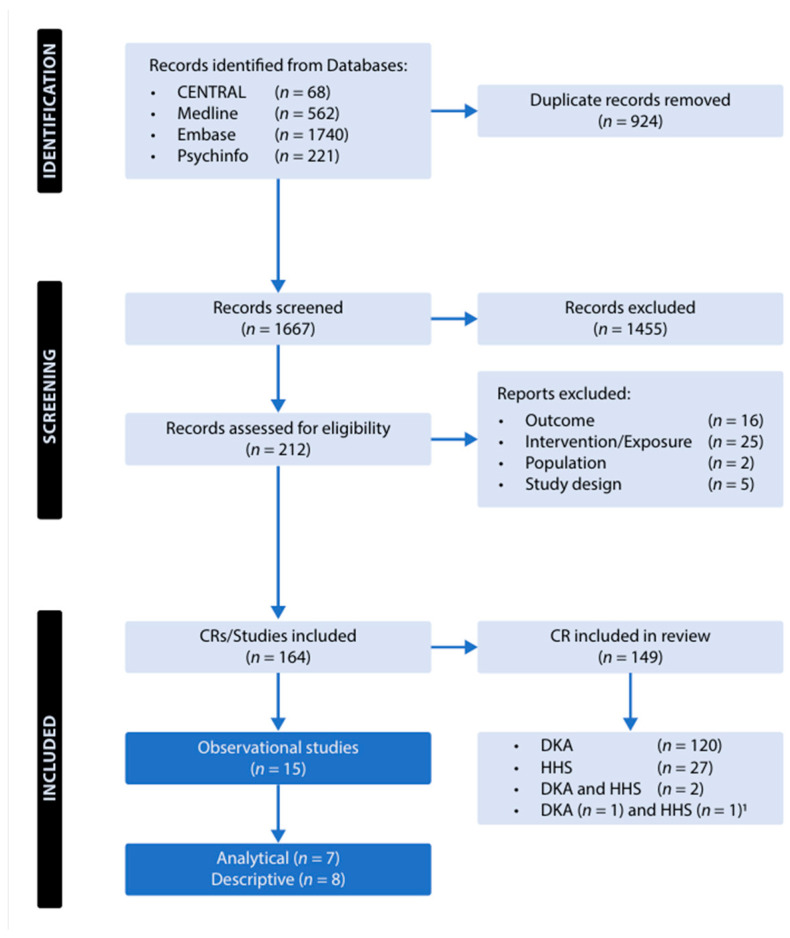
Flow chart for the selection of observational studies included in the systematic review. Abbreviations: CR, case report; CENTRAL, Cochrane Central Register of Controlled Trials; DKA, diabetic ketoacidosis; HHS, hyperglycemic hyperosmolar state. ^1^ Two additional case reports (one DKA and one HHS) were identified through supplemental searching outside the predefined search strategy.

The following bibliographic databases were searched to identify eligible observational studies of interest: MEDLINE, EMBASE, the Cochrane Central Register of Controlled Trials (CENTRAL), and PsycINFO through to 17 October 2025 (Figure 1). To complement the primary search, a secondary search consisting of hand-searching of reference lists and citation tracking was conducted. The search strategy, detailed in Supplemental Material I, was reviewed by a research librarian from the University of Ottawa’s Health Sciences Library to ensure comprehensive and systematic coverage of relevant literature. The methodology adhered to established standards for systematic reviews and followed PRISMA guidelines (Supplemental Material, Table S1).

### 2.2. Data Screening and Abstraction

EndNote (Clarivate Analytics, 2018, Version X9, available from https://endnote.com) [30] was used to remove duplicate records identified from the database search. This was followed by two levels of screening by two independent reviewers to determine study eligibility. Phase 1 involved screening of titles and abstracts (Supplemental Material II). Phase 2 involved full text screening of all records retained from Phase 1. Screening was performed using Distiller SR (Evidence Partners, Canada, ON, Canada, 2018) [31]. Pertinent data were abstracted from each eligible study using Excel. Subsequently, observational studies were categorized as analytical or descriptive.

For the purposes of this review, analytical studies included those presenting adjusted or unadjusted effect estimates, such as relative risk, odds ratios, proportional reporting ratio (PRR), and reporting odds ratio (ROR) using cohort or nested case-control study designs and pharmacovigilance databases such as the Food and Drug Adverse Event Reporting System (FAERS) and the Japanese Adverse Drug Event Report database (JADER). Descriptive studies focused primarily on data from hospital death certificates, bibliographic databases, or the FDA Safety Information and Adverse Event Reporting Program (FDA MedWatch) program. The following information was abstracted from studies that met the inclusion criteria: type/names of databases, patient population, study design, methods (overall sample size and study period, description of the statistical analysis, inclusion and exclusion criteria), information on exposure (class of APDs, individual APDs, treatment information such as duration, dose, and form; notes on exposure ascertainment); information on outcomes of interest and outcome ascertainment; results on demographic variables (age, sex, and ethnicity of study participants), adjusted/unadjusted effect estimates, or raw data (counts, exposed/non-exposed, events/nonevents), relevant covariates; and conclusions including strengths and limitations.

Data abstraction was completed by one of the two reviewers (NH), with duplicate data abstraction conducted on a 10% random sample of the studies as a quality control measure (NF).

### 2.3. Eligibility Criteria

#### 2.3.1. Inclusion Criteria

Observational studies were considered if they evaluated at least one of the two outcomes of interest, DKA or HHS, and evaluated at least one FDA-approved APD type as an exposure (Table 1). Although DKA may be classified as mild, intermediate or severe, this distinction was not observed in any of the observational studies that otherwise met our eligibility criteria. Similarly, although HHS can demonstrate variable severity, this was not also not discussed in the observational studies included in the present review. Studies in which hyperglycemic conditions, including DKA and HHS were pooled, were included. Both English and French abstracts were considered. There were no restrictions on location, indication of APD use, or patient age.

#### 2.3.2. Exclusion Criteria

Studies were excluded if the outcomes examined were not DKA or HHS, if the APD was never approved or was discontinued from use in the US, or if APDs were not suspected to be responsible for one of the outcomes. Meta-analysis, editorials, comments, literature reviews, and systematic reviews were not included. Case reports that were eligible for analysis were included in a second review [26].

### 2.4. Quality Assessment

The quality of each included observational study was assessed using the Office of Health Assessment and Translation (OHAT) risk of bias rating tool, which is applicable to diverse study designs. It comprises seven domains: selection bias, confounding bias, attrition/exclusion bias, detection biases for exposure and for outcome characterization, reporting bias, and other possible biases (National Toxicology Program (NTP), 2015) [33]. A three-tiered approach was used to determine the overall study quality according to the Handbook for Conducting a Literature-Based Health Assessment (NTP, 2019) [34]. Tier 1 qualifies as “definitely” or “probably” low risk for bias; Tier 3 qualifies as “definitely” or “probably” high risk of bias. Tier 2 included studies that could not be classified as low or high risk of bias. For spontaneous reporting analyses, including reports from the FDA MedWatch program, several domains were rated as high risk or were marked as not applicable due to the absence of verified exposure, verified outcomes, comparison groups, or follow-up. These pharmacovigilance analyses were included for consistency, and the ratings reflect limitations inherent to spontaneous reporting analyses rather than deficiencies in study methodology. Evaluation of the risk of bias was done by one of the two reviewers (NH) with the second reviewer (NF) verifying a 10% sample of the studies evaluated.

## 3. Results

### 3.1. Scope of Included Studies

The criteria for inclusion were met by 15 observational (seven analytical and eight descriptive) studies. The studies varied in scope with respect to the case definition, study population, exposure to APDs, reported outcomes, effect estimates, or descriptive analysis of the results. Table 2 summarizes the design of these studies. Suzuki et al. [35] did not find serious adverse DKA events between the two groups under study, intramuscular olanzapine and intramuscular haloperidol. This study is not included in the Tables.

### 3.2. Analytical Studies

Four of the analytical studies chose patients with schizophrenia as their study subjects [36,37,39,42]. Ramaswamy et al. (2007) [40] and Lipscombe et al. (2014) [38] selected patients who had received an APD. All analytical studies focused their analysis primarily on atypical APDs, except for Leslie and Rosenheck (2004), and Lipscombe et al. (2014), who also analyzed typical APDs as a class [37,38].

Henderson et al. (2007) [36] provided descriptive results for DKA and HHS but refocused their analysis on the incidence of new-onset diabetes mellitus presenting as DKA events and compared it to those with known DM status presenting as DKA for various atypical APDs, possibly because there were few (*n* = 4) HHS cases (Table 3). Three studies provided effect estimates for the association between antipsychotic medications and DKA using hazard ratios, attributable risks, or crude rates [36,39,40]. In the sole nested case-control study that satisfied inclusion requirements, Polcwiartek et al. (2017) [39] evaluated the odds ratio for this association. One study presented a pooled analysis of hyperglycemia [40], and although it was valuable, the interpretation of the reported association between APDs and DKA or HHS was challenging to decipher.

Two pharmacovigilance analyses using FAERS and JARED were included in this review. Liang et al. (2025) [41] assessed DKA adverse events associated with the use of quetiapine as a primary suspect drug using the US FDA’s FAERS database in comparison with three other atypical APDs: clozapine, olanzapine, and risperidone. Sugawara et al. (2023) [42] examined the efficacy of olanzapine and haloperidol in schizophrenia patients at the Tanzawa Hospital in Japan and reported no DKA events. This study was not included in the descriptive tables or the risk of bias quality control assessment.

#### Summary of Effect Estimates from Analytical Studies

The incidence of new DM presenting as DKA in a population of inpatients and outpatients diagnosed with schizophrenia in Boston, Massachusetts was 2.2% and 0.8% for clozapine and olanzapine, respectively, followed by risperidone at 0.2%. The authors also found a significant difference between clozapine and risperidone in the incidence of diabetes presenting as DKA (*p* < 0.0001) [36]. During the seven-year follow up, the authors did not find any significant differences in age, glucose levels at the time of DKA occurrence, or hemoglobin A1c (HbA1c) levels between patients with new-onset DM presenting as DKA when compared to those with known DM. The overall incidence of DKA in patients suffering from schizophrenia and related disorders was 1.67% of all DKA cases in the hospital.

Using the Department of Veteran Affairs administrative health records database, Leslie and Rosenheck (2004) [37] studied an older, largely male, outpatient, diabetes-naïve population diagnosed with schizophrenia, of whom 7.3% developed diabetes and 0.2% were hospitalized for DKA. The authors used Cox proportional hazard models to model the time to DM diagnosis and time to DKA hospitalization. Hazard ratios (HRs) for diabetes were highest with the use of clozapine (HR: 1.57, 95% confidence interval [CI]: 1.31–1.89) and olanzapine (HR: 1.15, 1.07–1.24), followed by treatment with quetiapine (HR: 1.20, 0.99–1.44) and risperidone (HR: 1.01, 0.93–1.10). These estimates were not significantly different from those for typical APDs, and the attributable risks of DM associated with atypical APD use were small, in the range of 0.05% for risperidone to 2.03% for clozapine. HRs for DKA hospitalization were larger than the DM model and were significant for clozapine at 3.75 (1.39–10.09) and olanzapine at 1.77 (1.05–2.98). However, attributable risks were relatively small and ranged from 0.004% for risperidone to 0.071% for clozapine.

Polcwiartek et al. (2017) [39] also evaluated this association in patients with schizophrenia who did not have diabetes and estimated odds ratios for atypical APDs as a class due to the low number of individual drugs. The odds ratio (OR) for the association between atypical APD exposure and DKA within three months prior to the event was 2.60 (95% CI: 1.06, 6.38) in the crude model. After adjusting for low or moderate/high Charlson comorbidity score within 1 year prior to the event, the OR was 2.58 (95% CI: 1.04, 6.40); OR was 2.65 (95% CI: 1.06, 6.59) and 4.06 (95% CI: 1.48, 11.11) after adjusting for diabetogenic and psychotropic co-medications within 3 months prior to the event, respectively. The authors concluded that most APDs (including typical agents) are associated with an increased risk of developing DKA, but the findings were not shown for individual drugs due to the limited number of cases.

Lipscombe et al. (2014) [38] pooled hyperglycemia, DKA, and HHS into one aggregate outcome and assessed this association in a newly exposed patient group (no APD prescription in the preceding 365 days before cohort entry date) in two sub-populations comprised of patients with or without diabetes. In patients with diabetes aged between 18 to 65 years of age, risperidone and olanzapine had crude incidence rates of 9.45 per 1000 persons (95% CI: 6.32–12.59) and 9.62 per 1000 (4.90–14.33), respectively. The crude event rates for risperidone and olanzapine in patients without diabetes were 0.36 per 1000 (0.22–0.50) and 0.51 per 1000 (0.30–0.73), respectively. After adjusting for relevant covariates, the hazard ratio for an olanzapine-related hyperglycemic event was 1.15 (0.71–1.86) overall, and 1.19 (0.52–2.72) in those with pre-existing DM; there was no significant difference in hyperglycemic events between new users of risperidone and olanzapine. In diabetic patients aged 66 years of age or more, the incidence rate was 7.07 per 1000 persons (6.00–8.14) for risperidone and 6.74 per 1000 persons (4.90–8.57) for olanzapine, respectively. The rates were much lower in non-diabetics at 0.85 per 1000 persons (0.67–1.02) for risperidone and 0.78 per 1000 persons (0.49–1.08) for olanzapine. The research group also completed a secondary nested case-control analysis for cases who experienced a hyperglycemic event to matched controls who did not. While the authors did not present quantitative results in their paper, they noted that there were no statistical differences in outcomes between current use of olanzapine, other atypical antipsychotics, or typical antipsychotics compared with current exposure to risperidone. Due to the lack of information, this secondary analysis was not included in the tables (Table 3; Supplemental Material Table S2).

Ramaswamy et al. (2007) [40] studied a retrospective cohort of patients receiving an atypical APD [39], the unadjusted risk of developing of DKA was 1.8 times greater with olanzapine treatment when compared to risperidone (*p* = 0.0096); this relative risk (RR) was reduced to 1.62 after adjustment for relevant covariates but remained statistically significant (*p* = 0.033). As shown in the multivariable logistic regression analysis, pre-existing diabetes prior to atypical APD use (OR: 9.64; 95% CI: 6.066–15.341), presenting with schizophrenia (2.216; 1.400–3.467) or being of African American descent (1.764; 1.037–2.944) are strong predictors of DKA. In contrast, sex, other psychiatric conditions such as bipolar disorder and depression, as well as other races were not significantly associated with DKA. The unadjusted risk of DKA was 10.7 per 10,000 patients for olanzapine, and 6.0 per 10,000 patients for risperidone. The risk estimates for quetiapine and clozapine should be interpreted with caution due to the low number of events (Table 3). Although exposure-window data was not discussed in this review, the authors noted that the risk of DKA increased with longer duration of olanzapine exposure.

Liang et al. (2025) [41] analyzed the US Food and Drug Administration Adverse Event Reporting System (FAERS) database to evaluate quetiapine-related DKA adverse events (*n* = 3046). The majority of cases were from the United States (*n* = 2913; 95.6%). Quetiapine showed the strongest disproportionality signal with an ROR of 31 (95% CI: 29.9–32.3), followed by olanzapine (15.2; 14.2–16.2). Both APDs demonstrated statistically significant disproportionality, yet the authors classified these as weak signals because the lower bound of the 95% CI was below their predefined threshold of 50. Clozapine and risperidone did not generate significant signals. Hospitalization was the most frequent severe ADR at 17.70% (*n* = 539), followed by death at 10.93% (*n* = 333).

Sugawara et al. (2023) [42] identified 55 cases of DKA in patients with schizophrenia in the Japanese Adverse Drug Event Report database. A disproportionality signal for DKA was detected for olanzapine, with adjusted ROR of 3.26 (95% CI: 1.87–5.66). Adjusted RORs were not statistically significant for other atypical APDs (risperidone, aripiprazole, and quetiapine) and typical APDs (chlorpromazine, haloperidol, levomepromazine, and paliperidone). In a multivariable logistic regression analysis, the authors reported that being male was associated with increased odds of DKA in relation to use of olanzapine (2.72; 1.07–6.90). Three patients (6%) died following the occurrence of DKA.

### 3.3. Descriptive Studies

The inclusion criteria were satisfied by a total of eight descriptive studies as described below. Ely et al. (2013) [43] examined Medical Examiner Records in New York City. For this analysis, death certificates and toxicology data from former psychiatric patients treated with atypical APDs were analyzed for electrolytes and glucose, including urine and blood tests, medical history, and toxicology results, which provided information on drug use and metabolic markers. A cross-sectional analysis of a prospective survey of young patients in Germany and Austria suffering with T1DM treated with typical and atypical APDs was conducted by Galler et al. (2015) [44] and compared those who were administered antipsychotic medicines to those who did not receive it. Jin et al. (2002) [45] used bibliographic databases and compared patient characteristics for those who developed new-onset DM only and those who developed DKA after initiation of atypical APD treatment. Koller et al. (2001; 2002; 2003; 2004) [46,47,48,49] used the FDA MedWatch Drug Surveillance Program, Medline, and selected abstracts from meetings to present descriptive statistics for hyperglycemia and associated exacerbations with an emphasis on acidosis and ketosis mainly, but without a consistent distinction between these conditions and DKA in all the results that were presented. The authors also noted the occurrence of HHS, but only provided sufficient details for four of the HHS cases, suspected to be induced by olanzapine [47] or quetiapine [49].

#### Summary of Descriptive Statistics

Descriptive observational studies included in this review provide additional evidence of a potential association between atypical APD use and hyperglycemic complications, mainly DKA (Table 4; Supplemental Material Table S3). Ely et al. (2013) [43] used New York’s City Office of Chief Medical Examiner Records (NYC OCME) for psychiatric patients treated with widely used atypical APDs in the United States, namely: quetiapine, olanzapine, risperidone, ziprasidone, and clozapine. Death occurred in ten patients with schizophrenia; three decedents had a combined diagnosis of schizophrenia and bipolar disorder, two patients were diagnosed with bipolar disorder, and two others with major depression. The authors concluded APD use was the primary cause of death in 16 of the decedent cases, while the immediate cause of death in all cases was DKA. While only ten deaths were formally attributed to treatment complication, Ely et al. (2013) [43] suggested that six additional deaths could be reasonably classified as treatment-related, given the potential for drug-associated adverse events. The detection of blood ketones for 17 decedents supported their conclusions.

A prospective analysis of a German/Austrian diabetes survey of patients who were 25 years of age or younger and diagnosed with T1DM indicated 0.48% of participants were treated with APDs. Among atypical drugs, risperidone was the most prescribed atypical APD (*n* = 122, 42%), followed by quetiapine (*n* = 19, 7%). For typical antipsychotic agents, pipamperone was the most prescribed typical APD at 10% (*n* = 29), followed by promethazine, prochlorperazine and haloperidol at 6% each *(n* = 18, 17 and 16, respectively). Poisson regression analysis, adjusted for age, sex, and diabetes duration, and diabetes center, for classes of drugs showed that the risk of DKA in patients undergoing with atypical APD treatment compared to patients who were not receiving these drugs, was 0.12 vs. 0.05 (*p* < 0.001) per patient-year of observation; the risk of DKA for patients receiving typical APD treatment was 0.13 vs. 0.05 (*p* < 0.001) per patient-year [44]. In contrast, adjusted linear regression analysis demonstrated a small effect of APDs on glycemic control. Atypical APD use was associated with a slightly higher hbA1c (8.7% vs. 8.4% *p* = 0.022), whereas typical APDs and APD use overall showed no significant differences.

Jin et al. [45] evaluated 45 published cases of new-onset DM (*n* = 26) or DKA (*n* = 19, 42%) following initiation of atypical antipsychotic agents. Clozapine and olanzapine were administered to 20 and 19 patients, respectively; quetiapine and risperidone were administered to 3 patients each. Comparison of the DM and DKA cohorts indicated that that patients presenting with DKA were younger, often female, less overweight at baseline, and had higher blood glucose levels. There were no differences between the groups in terms of ethnicity, family history of DM, duration of APD exposure, and weight gain.

A series of analysis of the FDA MedWatch Drug Surveillance Program by Koller et al. evaluated the association between four different atypical APDs and hyperglycemia, including acidosis, ketosis, DKA, and HHS. As mentioned previously, the authors did not consistently distinguish between different states of diabetic complications or provide sufficient information on DKA or HHS cases. Based on their findings, they suggested a potential association between certain atypical APDs—namely clozapine, risperidone, olanzapine, and quetiapine—and hyperglycemia [44,45,46,47,48] (Table 4; Supplemental Material Table S3).

### 3.4. Study Quality and Risk of Bias

All studies included in this review were subject to an assessment of study quality and risk of bias using the OHAT tool, except for the analysis by Suzuki et al. (2013) [35]. Their analysis did not find any association between serious adverse DKA events between the olanzapine and haloperidol-treated elderly patients with agitated schizophrenia.

Three analytical studies [38,39,40] were rated as having “definitely or probably” low risk of bias for the three key criteria (confounding bias and detection biases for exposure and outcome). Two analytical studies [36,37] and two descriptive studies [44,45] were classified in Tier 2 because they did not meet the criteria for the first or third tier. Two pharmacovigilance analyses using spontaneous reporting data from the FAERS and JADER databases were classified as Tier 3, reflecting a high risk of bias inherent to spontaneous reporting systems [41,42]. Five descriptive studies were classified as Tier 3, with a “probably or definitely” high risk of bias [43,46,47,48,49] (Supplemental Material Table S4).

## 4. Discussion

The present review provides an overview of observational studies that evaluated the potential association between treatment with APDs and serious complications of diabetes, specifically DKA and HHS. Henderson et al. (2007) [36] reported an incidence of new-onset DM presenting as DKA that was 10 times higher than that of the general population in patients with schizophrenia treated primarily with clozapine, olanzapine, and risperidone (14.9 vs. 1.4 cases per 10,000 patient years, *p* < 0.00001). Elevated levels of HbA1c indicate that DM may have been undiagnosed prior to DKA events observed in this study. These findings are corroborated by Lipscombe et al. (2014) [38], who found that while the risks remain low, those with pre-existing diabetes remain at greater risk of developing diabetic emergencies after initiation of APD treatment. Using data from a nationwide German/Austrian diabetes survey, Galler et al. (2015) [44] concluded the rate of acute DKA in young patients was higher in those treated with APDs when compared to patients who did not receive these medications. While these findings supplement and support those from our recently published case report/case series [26], many observational analyses on the incidence of DKA and HHS do not consider the indication of antipsychotic medication use. In a multicenter prospective initiative involving patients with diabetes across 437 centers in Germany, Austria, Switzerland, and Luxembourg, Tittel et al. (2020) [50] evaluated the occurrence of DKA and HHS in patients with pre-existing diabetes. Findings indicated that being diagnosed with T1DM and being of younger age were strong indicators for diabetic emergencies associated with DKA and HHS, although both conditions also occurred in those diagnosed with T2DM. Patients who experienced DKA and HHS had worse metabolic control, and both conditions were linked to heavy alcohol drinking and comorbidities such as dementia, stroke, chronic kidney disease, and depression. A recent retrospective analysis of a diabetic cohort based in Ethiopia corroborates that the incidence of hyperglycemic emergencies is higher in those diagnosed with diabetes [51]. It is important to note that while diabetic duration is a strong predictor of developing complications, findings across studies are inconsistent [52,53]. It would be worth investigating this predictor in future observational studies.

Leslie et al. (2004) [37] concluded that despite the low absolute risk, the severity of the event justifies a closer examination of the attributable risk for clozapine and risperidone monotherapy in diabetes-naïve older male populations with schizophrenia. Ramaswamy et al. (2007) [40] estimated that the attributable risk of DKA was 1.62 times higher with olanzapine monotherapy compared to risperidone monotherapy, irrespective of DM diagnosis (*p* = 0.033). A descriptive analysis of a diabetes-naïve outpatient population established an association between clozapine use and DKA early in the course of treatment; notably, the administration of antidiabetic medications was negatively correlated with DKA (*p* < 0.0001) [54]. However, this protective effect should be interpreted with caution, since it is partially due to sampling bias and new-onset occurrence of DKA in patients not previously diagnosed with diabetes [54].

While antidiabetic agents such as metformin and sodium-dependent glucose transporter (SGLT2) are effective glucose lowering agents [55], they act by different mechanisms to achieve their intended result. Metformin improves insulin sensitivity [56] and reduces hepatic glucose production through inhibition of gluconeogenesis [56,57]; SGLT2 inhibitors block reabsorption of glucose in the proximal tubule of the kidney, thus causing glycosuria, and increase renal glucose excretion [58] so that these medicines can protect against kidney and heart failure [59].

In recent years, however, several case reports attributed the occurrence of euglycemic DKA (defined as blood glucose < 250 mg/dL and presence of severe metabolic DKA) with treatment with SGLT2 inhibitors in some patients with T2DM [60,61,62,63,64]. This prompted the issuance of a safety communication issued by the US Food and Drug Administration (FDA) in 2015 following an FAERS analysis of 20 cases of acidosis in patients taking SLGT-2 inhibitors [65]. This was further corroborated by more recent analysis using FAERS, which supported the FDA warnings [66,67,68]. Subsequently, the American Association of Clinical Endocrinologists (AACE) and American College of Endocrinology (ACE) issued a position statement on the association of SGLT-2 inhibitors and DKA [69,70]. Similarly, the European Medicines Agency (EMA) published recommendations to minimize the risk of DKA in patients treated with SGLT2 inhibitors [70,71]. Morace et al. (2024) [70] noted that physicians and patients must be cognizant of the risk of developing euglycemic DKA with the use of SGLT2s in T1DM and T2DM [72,73]. Regular monitoring of precipitating factors, including co-existing conditions, infection, reduced daily insulin dose in insulin-treated patients, and surgery would help mitigate the risks of developing DKA with the use of SGLT2 inhibitors when used as authorized and in accordance with treatment guidelines [70]. Treatment can be reinstated once ketone values normalized and the patient’s condition stabilized.

The above discussion draws attention to the need to consider potential drug–drug interactions when assessing the risk of diabetic complications in diabetic patients taking APDs and treated with different antidiabetic medications. A review by Vasiliu (2023) [74] concluded that further pre-clinical and clinical research is needed to understand possible drug–drug interactions following the concomitant treatment with APDs and SGLT2s in patients presenting with severe mental illness. Although co-medication with SGLT2 and metformin has been associated with beneficial metabolic effects in some patients with T2DM, there is limited data on the use of SGLT2 as second-line treatment in patients with diabetes mellitus who are treated with olanzapine and clozapine [74,75,76].

Koller et al. evaluated spontaneous reporting of hyperglycemic adverse events, including DKA and HHS, and the use of clozapine [46] and olanzapine [47] in the US FDA MedWatch Surveillance Program. These descriptive studies are informative and provide additional weight to existing evidence supporting an association between APDs and DKA and HHS. However, for the purpose of this review, these findings cannot be interpreted unequivocally, as the authors do not always differentiate between distinct states of hyperglycemia, including acidosis, ketosis, and DKA when presenting their results. We recently evaluated reports of diabetic ketoacidosis (DKA) associated with atypical and typical APD use, where certain APDs were identified as primary or secondary suspect drugs, submitted to the US FDA FAERS between January 2000 and December 2022 [77]. Notably, 12.5% of DKA adverse event (AE) reports listed atypical APDs as the primary suspect drug, whereas reports involving typical APDs were scarce. Disproportionality analysis indicated that olanzapine and quetiapine, the most frequently reported atypical APDs, generated strong safety signals relative to all other drugs (olanzapine: PRR 13.2, 95% CI 12.4–14.2; quetiapine: 11.8, 11.1–12.5). A little less than half of all reports originated from the United States (45.4%), with hospitalization being the most frequently reported clinical outcome among DKA-related AEs. These findings align with the pharmacovigilance analysis by Liang et al. (2025) [41], which specifically focused on quetiapine-associated DKA. In that study, quetiapine demonstrated the strongest reporting signal for DKA, followed by olanzapine. The majority of reports also originated from the United States, and hospitalization was the most common severe adverse drug reaction outcome (17.7%). In a separate analysis, we evaluated clinical evidence from published case reports describing adverse drug reactions, specifically DKA and HHS, associated with the use of both first- and second-generation antipsychotics. The most common psychiatric diagnosis was schizophrenia (*n* = 77, 51%), followed by bipolar disorder (*n* = 26, 17.2%). Olanzapine was associated with the highest number of DKA cases (*n* = 53, 43.1%), followed by clozapine (*n* = 24, 19.5%). Among the 12 reported fatalities, olanzapine was implicated in four DKA cases and one HHS case, clozapine in one DKA case, and quetiapine in one DKA case and one HHS case [26]. Taken together, these pharmacovigilance and clinical analyses highlight a potential safety signal for DKA associated with atypical antipsychotic drugs, particularly quetiapine and olanzapine. While these findings do not establish causality, they underscore the need for further clinical and epidemiological investigation.

Sugawara et al. (2023) [42] demonstrated that treatment with olanzapine was associated with the development of DKA among patients with schizophrenia. Consistent with these findings, our recently published systematic review indicated that 50.9% of olanzapine-related DKA cases occurred in patients with schizophrenia, while 87.5% of clozapine-related DKA cases were reported in this population, with a higher proportion observed among male patients [26]. Sugawara and colleagues further reported that male patients were associated with an increased risk of DKA among patients treated with olanzapine (aROR 2.72 (1.07–6.90). Although the study designs differ, these findings suggest that male patients with schizophrenia treated with olanzapine or clozapine may be at increased risk for DKA. Clinicians should therefore remain vigilant, and proactive monitoring and management of glucose levels may help reduce the occurrence of antipsychotic-associated DKA in the treatment of schizophrenia.

A review by Cohen et al. (2012) [78] compared one-year incidence rates and mortality rates due to DKA adverse events involving use of clozapine. The incidence of DKA was 1.2–3.1%, with a case fatality rate of 20–31%. The high risk of mortality renders DKA one of the riskiest side effects, even with low dose and early course of treatment with clozapine [54].

In addition to synthesized evidence from case reports, evaluation of rare but life-threatening conditions such as DKA and HHS using prospective or retrospective cohorts is necessary. Randomized controlled trials (RCTs) cannot support hypothesis testing for every conceivable research question, particularly those involving life-threatening conditions. To our knowledge, The Ziprasidone Observational Study of Cardiac Outcomes (ZODIAC), open-label RCT trial study led by Strom et al. (2011) [79] is the only RCT that evaluated the association between APD use and DKA as a secondary endpoint. Two treatment groups—ziprasidone or olanzapine—were compared in schizophrenia patients enrolled from eighteen different countries. Hospitalization rates for DKA in both treatment groups were low, consistent with the reported medical history of T1DM in both groups at baseline also being low (<3%). In a primary intent-to-treat analysis, there were no differences between the two treatment groups with respect to DKA (Relative risk (RR): 1.00, 95% CI: 0.29–3.45). A secondary analysis of person–time on assigned treatment provided similar results (1.11 (0.32–3.85). It is recommended that these results be interpreted with caution since they are underpowered due to the small sample size in each treatment group.

Most studies included in this review cautioned against establishing a causal link between APD use and DKA or HHS, since strong and defensible assumptions are essential to infer causality from observational studies [80]. Although adjusting for measured and known confounders is possible, a significant effect estimate does not imply that a drug is responsible for the outcome being examined. Therefore, when assessing the relationship between drug exposures and life-threatening conditions, it is important to evaluate the weight of evidence for causality to properly interpret the observed associations between antipsychotic use and DKA or HHS. This is especially important when the underlying biological mechanisms of actions linking exposure and outcome are not fully understood.

Antipsychotic medication users are at elevated risk of developing hyperglycemia due to several risk factors, including genetic and lifestyle health determinants [1]. In a second scenario proposed by the author, antipsychotics may have a causal effect and raise the risk of this metabolic disorder through their direct impact on body weight, insulin resistance and impaired insulin secretion. Holt (2019) [1] suggests these two possibilities are not mutually exclusive. These scenarios can be extrapolated to serious diabetic complications such as DKA and HHS. Consideration also needs to be given to off-label use of APDs and non-adherence to treatment. APDs are first-line treatments for severe mental illnesses such as schizophrenia, yet off-label use of these agents to treat conditions such as mood and anxiety disorders remains common across all age categories [81]. As mentioned earlier, the seriousness of the mental health conditions these medications treat can lead to reduced adherence with clinical treatment plans [18]. These considerations, together with dose-dependence and biological plausibility, make it difficult to evaluate whether observed relationships represent true causality or merely an association. Although we adapted the scenarios presented by Holt (2019) [1] to provide a high-level overview of the potential association and inference of causality of APD use and hyperglycemic emergencies [77], we are fully cognizant more research is necessary to understand the etiology of DKA and HHS.

### 4.1. Strengths

To our knowledge, this is the first comprehensive review of descriptive and analytical observational research that seeks to clarify the relationship between APD medication use and hyperglycemic complications, namely DKA and HHS. As was evident in our review on case reports [26], there is a need for additional observational studies to provide robust effect estimates for this newly emerging and complex association.

### 4.2. Limitations

The heterogeneity in scope and methodological design of the independent studies included in this review made it challenging to conduct a meta-analysis to quantitatively summarize overall findings. To date, there are a limited number of observational studies that evaluated APD exposure and its association with diabetic emergencies. This may be due to various reasons: (1) off-label use of APDs adds another layer of complexity to the interpretation of effect estimates, particularly since information on indication of use is not always available; (2) non-compliance to treatment can substantially affect true differences between groups, biasing results toward the null due to reduce statistical power; and (3) gaps in data availability and collection, particularly the lack of relevant biomarkers, can have a strong impact on data quality. These limitations make it difficult to draw firm conclusions on the strength of evidence for a causal relationship between APDs and DKA and HHS.

## 5. Conclusions

Collectively, these observational studies provide further evidence supporting an association between atypical antipsychotic drug use and DKA. These findings also suggest that, as a class, typical antipsychotic drugs are associated with a higher risk of DKA. Future research should include detailed prospective and retrospective analytical studies to examine this association in more depth. At this point in time, there is insufficient evidence to draw conclusions regarding HHS. Nonetheless, although HHS is less common than DKA, it is important to evaluate this outcome in relation to antipsychotic use. As the mechanisms leading to these complications are multifactorial and complex, preventative measures would allow for an early recognition of symptoms and prompt treatment.

## Figures and Tables

**Table 1:** List of Atypical and Typical Antipsychotic Drugs Included in the Search Strategy ^1^.

Atypical APDs	Typical APDs
Aripiprazole	Chlorpromazine
Asenapine	Droperidol
Cariprazine	Fluphenazine
Clozapine	Haloperidol
Iloperidone	Loxapine
Lurasidone	Molindone
Olanzapine	Perphenazine
Paliperidone	Pimozide
Pimavanserin	Prochlorperazine
Quetiapine	Thiothixene
Risperidone	Thioridazine
Ziprasidone	Trifluoperazine

^1^ Source: [32].

**Table 2:** Study Design for observational studies on the association between antipsychotic drugs and diabetic ketoacidosis or hyperosmolar hyperglycemic state.

Author (year) and Study Location	Study Design
Analytical Epidemiological Studies	
Henderson et al. (2007) Boston Massachusetts, United States of America [36]	• Retrospective cohort based on Research Patient Data Registry (RPDR) (1 January 1995 to 31 December 2001) • 819,308 inpatients and outpatients who received care at the Massachusetts General Hospital during the 7-year period.
Leslie & Rosenheck (2004) United States of America [37]	• Retrospective Cohort based on administrative Data from the Department of Veterans Affairs (June 1999 to September 2001) • 73,946 patients with schizophrenia with stable regimen of APD MT
Lipscombe et al. (2014) Canada, United Kingdom [38]	• Multicenter retrospective cohort based on: ► Administrative health data from 7 Canadian provinces (British Columbia, Alberta, Saskatchewan, Manitoba, Ontario, Quebec, and Nova Scotia) ► Administrative data from the UK United Kingdom renamed Clinical Practice Research Datalink (CPRD) (1 April 1998–31 March 2010) ● 725,489 patients newly treated with an APD
Polcwiartek et al. (2017) Denmark [39]	• Retrospective nested case-control based on nationwide healthcare registers: ► The Danish Civil Registration System from 1968—data on birth date, sex and migrations ► The Danish Psychiatric Central Research Register from 1969—data on psychiatric admissions and diagnoses ► The Danish National Patient Register from 1977—data on admissions and diagnoses for physical conditions ► The Danish National Prescription Register from 1995—data on prescription-based medications ► The Danish Register of Causes of Death from 1970—data on mortality. (1 January 1995 to 31 December 2014) • 29,955 patients with SCZ in a diabetes-naïve population
Ramaswamy et al. (2007) California, United States of America [40]	• Retrospective cohort based on California Medicaid data (July 1997 to September 2000) • 141,286 patients receiving an atypical APD
Analytical Pharmacovigilance Studies	
Liang et al. (2025) Zhejiang, China [41]	• Retrospective pharmacovigilance disproportionality analysis based on the US Food and Drug Administration Adverse Event Reporting System (FAERS) (Q1 of 2004 to Q4 of 2024) • 65,536 ADRs with quetiapine as the primary suspect drug
Sugawara et al. (2023) Japan [42]	• Retrospective pharmacovigilance disproportionality and cased control analyses based on the Japanese Adverse Drug Event Report (JADER) (April 2004 and March 2021) • 692,154 with AE • 1399 olanzapine treatment cases (case-control)
Descriptive Studies	
Ely et al. (2013) New York City, New York, United States of America [43]	• Retrospective cohort based on the New York City Medical Examiner Records (January 2005 to December 2009) • 20 deaths in psychiatric patients treated with atypical APDs.
Galler et al. (2015) Germany, Austria [44]	• Cross-sectional (registry-based) on the Nationwide German/Austrian Diabetes Survey (DPV) (1995 to March 2013) • 60,162 young patients with T1DM, <25 years of age
Jin et al. (2002) (N/A), multiple countries [45]	• Retrospective case series based on Medline, PsycINFO (1980–2001) • 45 cases of new-onset DM and atypical APD treatment
Koller et al. (2001, 2003, 2004), Koller & Doraiswamy 2002) (United States & International Sources) [46,47,48,49]	• Retrospective pharmacovigilance survey of spontaneously reported AE based on FDA MedWatch Drug Surveillance Program. • Other Sources: ▪ Published cases identified in MEDLINE. ▪ Abstracts: ► National endocrinology and diabetes meetings [46] ► National psychiatry meetings [47,48,49] ▪ Drug Utilization Data: IMS health National Prescription Audit Plus and the IMS National Disease and Therapeutic Index. Koller et al. (2001) [46] • FDA MedWatch: January 1990–February 2001. • Medline: January 1985 to February 2001. • 384 cases of clozapine-associated diabetes or hyperglycemia Koller and Doraiswamy (2002) [47] • FDA MedWatch: January 1994 to May 2001 • MEDLINE: Through May 2001. • 237 cases of olanzapine-associated diabetes or hyperglycemia Koller et al. (2003) [48] • FDA MedWatch: ► February 1993 to February 2002 (risperidone) ► Late 1970s–February 2002 (haloperidol) • MEDLINE: Through February 2002. • 168 Risperidone and/or haloperidol treated patients with or without diabetes or hyperglycemia Koller et al. (2004) [49] • FDA MedWatch: 1 January 1997 to 31 July 2002 • MEDLINE: January 1997–July 2002 • 46 reports of quetiapine-treated patients

Abbreviations: AE; Adverse Event; ADR, Adverse Drug Report; APD, Antipsychotic Drug; DM, Diabetes Mellitus; FDA, Food and Drug Administration; MT, Monotherapy; SCZ, Schizophrenia.

**Table 3:** Characteristics of analytical observational studies on the association between antipsychotic drugs and diabetic ketoacidosis or hyperosmolar hyperglycemic state ^1,2^.

Author (year)	Exposure/Outcome	Descriptive Statistics	Effect Estimates		
			Unadjusted		Adjusted
**Analytical Epidemiological Studies**					
Henderson et al. (2007) [36]	**Exposure:** Atypical APDs: ▪ Olanzapine ▪ Clozapine ▪ Quetiapine ▪ Risperidone ▪ Ziprasidone **Outcome:** • Incidence of DKA or HHS in new-onset or existing DM, per 10,000 patient years	**During 7 year period** **Total number of patents with SCZ or SZA:** *n* = 4850 • Patients who presented with DKA: 23 **New-onset DM Existing DM** DKA: 11 DKA: 8 HHS: 2 HHS: 2 **Mean BG mg/dL** 795 ± 328 690 ± 158 (*p* = 0.81) **HbA1c (%):** 13.1 11.2 (*p* = 0.20) • New onset DM presenting as recurrent DKA: 3	**During 7-year period:** SCZ patients: *n* = 53,942 (6.6%) **Incidence of DKA in SCZ/SZA patients:** 19/1132 (1.67%) **Incidence of DKA per 10,000 patient years:** • SCZ/SZA patients without a prior diagnosis of DM: 14.93 • Massachusetts General Hospital general population: 1.98 (*p* < 0.000001) ► >10-fold higher in SCZ/SZA patients **New-onset DM presenting as DKA** Incidence for APDs used to treat SCZ/SZA over a 7-year period: *n* =; % • Clozapine 226; 2.2 • Olanzapine 776; 0.8 • Risperidone 585; 0.2 • No incidence for: • Quetiapine: 479 • Ziprasidone (57) • Clozapine vs. risperidone (*p* < 0.00001). • Clozapine vs. olanzapine (*p* = 0.117) • Olanzapine vs. risperidone (*p* = 0.18) • Clozapine and olanzapine (*p* = 0.117) • Olanzapine and risperidone (*p* = 0.18) • The inclusion of HHS cases would increase the incidence of olanzapine to 1.0%.		N/A
Leslie & Rosenheck (2004) [37]	**Exposure:** Atypical APDs (3-months period): • Clozapine • Risperidone • Olanzapine • Quetiapine • Typical APDs as a class **Outcome:** DKA	Patients with SCZ on stable APD MT without DM: *n* = 56,849 • Hospitalization for DKA: *n* = 88 (0.2%)	N/A		**DKA model:** APD, HR (CI) • Clozapine: 3.75 (1.39–10.09) • Olanzapine: 1.77 (1.05–2.98) **Attributable risks:** • Risperidone: 0.004% • Clozapine: 0.071% • HR for DKA: no statistical significance when analysis comprised DM patients only.
Lipscombe et al. (2014) ^1^ [38]	**Exposure:** • New exposure: no prescription to an APD in the preceding 365 days. Atypical APDs: • Risperidone (reference) • Olanzapine (primary comparator); • Collective exposure: clozapine, quetiapine, ziprasidone, paliperidone, aripiprazole • Collective exposure to typical APDs ^1^. **Outcome:** • Hospital admission due to hyperglycemic emergency: • Pooled Hyperglycemia, DKA and HHS ▪ within 365 days following drug initiation (cohort entry date)	**Pooled hyperglycemic emergencies: hyperglycemia, DKA and HHS** **Number and proportions of patients according to diabetes status: *n*; %** **Age 18–65 years** • Diabetes Status: 16,793; 5.2 • No diabetes: 307,719; 94.8 **Age ≥ 66** • Diabetes Status: 75,970; 18.9 • No diabetes status: 325,007; 81.1 **Age 18–65 years** • Risperidone: 76,212; 23.5 • Olanzapine: 47,699; 14.7 • Other atypical: 123,971; 38.2 • Typical: 76,630; 23.6 • Overall: 324,512 **Age ≥ 66** • Risperidone: 158,019; 39.4 • Olanzapine: 52,351; 13.1 • Other atypical: 75,514; 18.8 • Typical: 115,091; 28.7 • Overall: 400,977	**Crude hyperglycemic events (pooled) rates according to age group, diabetes status and drug exposure (per 1000 person years, 95% CI)** **Age group 18–65 years:** **Diabetes (*n* = 16,793)** • Risperidone: 9.45 (6.32–12.59) • Olanzapine: 9.62 (4.90–14.33) • Other atypical: 11.71 (9.01–14.41) • Typical: APDs: 14.20 (10.23–18.18) • Overall: 11.49 (9.78–13.21) **No diabetes** (*n* = 307,724) • Risperidone: 0.36 (0.22–0.50) • Olanzapine: 0.51 (0.30–0.73) • Other atypical: 0.30 (0.20–0.41) • Typical: APD 0.46 (0.30–0.63) • Overall: 0.39 (0.31–0.46) **Age group 66+ years:** **Diabetes:** (*n* = 75,970) • Risperidone: 7.07 (6.00–8.14) • Olanzapine: 6.74 (4.90–8.57) • Other atypical: 4.98 (3.76–6.20) • Typical 7.35 (5.95–8.76) • Overall: 6.64 (5.98–7.30) **No diabetes** (*n* = 325,007) • Risperidone: 0.85 (0.67–1.02) • Olanzapine: 0.78 (0.49–1.08) • Other atypical: 0.67 (0.45–0.90) • Typical: 1.03 (0.79–1.27) • Overall: 0.85 (0.74–0.97)		**Fixed-effect-meta-analyses:** • Risperidone: 1.00— **Age 18–65 years** **Overall HR (95% CI)** • Olanzapine: 1.15 (0.71–1.86) • Other Atypical: 0.94 (0.64–1.38) • Typical: 0.95 (0.61–1.54) **Diabetes HR 95% CI** • Olanzapine: 1.19 (0.52–2.72) • Other Atypical: 0.89 (0.45–1.68) • Typical: 1.09 (0.61–1.95) **Age ≥ 66** **Overall HR (95% CI)** • Olanzapine: 1.13 (0.87–1.48) • Other atypical: 0.69 (0.53–0.90) • Typical: 1.03 (0.73–1.45) **Diabetes HR (95% CI)** • Olanzapine: 1.12 (0.80–1.56) • Other atypical: 0.68 (0.49–0.95) • Typical: 0.62 (0.41–0.95)
Polcwiartek et al. (2017) ^2^ [39]	**Exposure** (within 3 months prior to event): • At least one prescription of an APD. • FGA (low-potency, mild-potency and high-potency) •SGAs: Amisulpride, aripiprazole, asenapine, clozapine, olanzapine, paliperidone, quetiapine, risperidone, sertindole, ziprasidone **Outcome:** DKA	**Characteristics of DKA cases and their matched controls:** **Cases** (*n* = 28) APD Exposure (within 3 months prior to event): *n*; % • Any APD: 20; 71.4 • APD polypharmacy: 2; 7.1 **FGAs** • Low-potency: 3; 10.7 • Mid-potency: 2; 7.1 • High-potency: 1; 3.6 **SGAs** • Amisulpride: 1; 3.6 • Clozapine: 4; 14.3 • Olanzapine: 3; 10.7 • Quetiapine: 4; 14.3 • Risperidone: 4; 14.3 **Controls** (*n* = 137) • APD exposure (within 3 months prior to event): *n*; % • Any APD: 67; 48.9 • APD polypharmacy: 2; 20.4 **FGAs** • Low-potency: 16; 11.7 • Mid-potency: 15; 10.9 • High-potency: 5; 3.6 **SGAs** • Aripiprazole: 3; 2.2 • Clozapine: 7; 5.1 • Olanzapine: 31; 22.6 • Paliperidone: 2; 1.5 • Quetiapine: 8; 5.8 • Risperidone: 13; 9.5 • Sertindole: 1; 0.7 • Ziprasidone: 1; 0.7	**Crude model (matched by age, sex, and year of schizophrenia onset):** • Reference: No APD • OR: 2.60 (1.06–6.38) (*p* = 0.036)		**Logistic Regression Models estimating the association between APD exposure (pooled) within 3 months prior to DKA:** • Reference: No APD • OR (95% CI), *p* value) **Model 1:** 2.58 (1.04, 6.40) (*p* = 0.041) **Model 2:** 2.65 (1.06, 6.59) (*p* = 0.037) **Model** 4.06 (1.48, 11.11) (*p* = 0.006) • ORs for individual APDs were not estimated due to low number of cases.
Ramaswamy et al. (2007) [40]	**Exposure:** • Risperidone • Olanzapine **Outcome:** • DKA	**Atypical APDs** **• Risperidone: *n*** Initial users: 51,330 DKA: 31 **• Olanzapine** Initial users: 51,302 DKA: 55 **•** Analysis excluded clozapine and quetiapine due to small sample size. **Clinical characteristics of patients with attributable DKA:** DM diagnosis prior to DKA (*n*; %): **• Olanzapine:** Yes: 36; 65.5 No: 19; 34.4 **• Risperidone:** Yes: 24; 77.4 No 7; 22.6 (*p* < 0.2461) **DM prior to APD initiation:** **• Olanzapine:** Yes: 23; 41.8 No: 32; 58.2 **• Risperidone:** Yes: 18; 58.1 No: 13; 41.9 (*p* < 1.000)	• Unadjusted risk of DKA was 1.8 times higher with olanzapine than risperidone (*p* = 0.0096). Attributable cases of DKA and the unadjusted risk (*n*; Risk per 10,000): • Clozapine: 1; 12.25 • Olanzapine 55; 10.72 • Risperidone: 31; 6.04 • Quetiapine: 4; 5.64		**Risk for DKA event:** OR (95% CI) • Olanzapine MT (vs. risperidone): 1.623 (1.047–2.560) (*p* = 0.033) • SCZ (vs. no SCZ): 2.216 (1.400–3.467) (*p* = 0.001) • African American (vs. white): 1.764 (1.037–2.944) (*p* = 0.032) • Presence of DM prior to APD use (vs. no DM prior to APD use): 9.643 (6.066–15.341) (*p* < 0.0001)
**Analytical Pharmacovigilance Studies**					
Liang et al. (2025) Zhejiang, China [41]	**Exposure:** Primary suspect drug Atypical APD: • Quetiapine Other atypical APDs: • Clozapine • Olanzapine • Risperidone **Outcome** (identified using PTs in MedDRA SMQ for hyperglycemia and new-onset DM): • DKA • Euglycemic DKA	**Characteristics: *n*** • ADRs with DKA and Quetiapine: 14,355 • Quetiapine as primary suspect drug: 3046 • Positive signals met AE criteria: 1236; 23.7% • Death: 333; 10.93 Top 5 SOCs: PT count; % • Psychiatric disorders 234; 18.93 • Nervous system disorders 169; 13.67 • Investigations 115; 9.3 • Injury: 62; 5.02 • Metabolism and nutrition disorders:52; 4.21	**PTs for DKA-related events (SOC: Metabolism and nutrition disorders)**		
			**PT** Ketoacidosis DKA Euglycemic DKA	**ROR (95% CI)** 68.76 (64.83–72.92) 19.78 (18.76–20.84) NR	**PRR (χ^2^)** 67.33 (73,755.21) 19.37 (24,733.80) NR
			**Correlation between quetiapine and DKA: Comparison to clozapine, olanzapine, and risperidone**		
			***n***		
			**Atypical APD** Quetiapine Clozapine Olanzapine Risperidone	**ROR, (ROR025-ROR975)** 3046 31.05 (29.87–32.27) 93 0.61 (0.50–0.75) 960 15.16 (14.21–16.19) 172 1.41 (1.21–1.64)	**Signal Strength** Weak No signal Weak No signal
Sugawara et al. (2023) [42]	**Exposure:** Atypical APDs: • Olanzapine, • Risperidone, • Aripiprazole, • Quetiapine Typical APD: • Chlorpromazine, • Haloperidol, • Levomepromazine, • Paliperidone **Outcome** (identified using PTs in MedDRA): • DKA	**Characteristics: *n*** • AEs reports with SCZ: 7435 • APD reports with DKA: 55 • Deaths: 5.5; 6% **Atypical APDs: *n*** • Olanzapine: 24 • Risperidone: 13 • Aripiprazole: 12 • Quetiapine: 9 **Typical APDs: *n*** • Chlorpromazine: 6 • Haloperidol: 6 • Levomepromazine: 5 • Paliperidone: 4		**Spontaneous Reporting—Signal detection** **APD: aROR (95% CI)** • Olanzapine: 3.26 (1.87–5.66). • Risperidone: 0.83 (0.44–1.56) • Aripiprazole: 1.03 (0.54–1.96) • Quetiapine: 1.54 (0.75–3.18) • Chlorpromazine: 1.19 (0.50–2.83) • Haloperidol: 0.94 (0.40–2.21) • Levomepromazine: 0.90 (0.36–2.30) • Paliperidone: 0.77 (0.27–2.17) **Case-Control Analysis—Multivariable logistic regression analysis** • Olanzapine-treated cases (*n* = 1399); male sex: 2.72 (1.07–6.90)	

Abbreviations: AE; Adverse Event; ADR, Adverse Drug Events; aROR, Adjusted Reporting Odds Ratio; APD, Antipsychotic Drug; BG, Blood Glucose; CI: Confidence Interval; DKA, Diabetic ketoacidosis; DM, Diabetes Mellitus; FGA, First-Generation Antipsychotic; HbA1c, Hemoglobin A1c; HHS, Hyperglycemic Hyperosmolar State; HR, Hazard Ratio; MedDRA, Medical Dictionary for Regulatory Activities; MT, Monotherapyio; OR, Odds Ratio; PRR, Proportional Reporting Ratio; PT, Preferred Term; SOC, System Organ Classification; SCZ, Schizophrenia; SD, Standard Deviation; SGA, Second-Generation Antipsychotic; SMQ, Standardized MedDRA Query; SZA, Schizoaffective Disorder; χ^2^, chi-square. Symbols: > more than; ≥, greater than or equal to; < less than; ≤, less than or equal to. ^1^ Lipscombe et al. (2014) [38] analyzed the collective exposure to typical APDs (chlormezanone, chlorpromazine, chlorprothixene, flupenthixol, fluphenazine, fluspirilene, haloperidol levomepromazine/methotrimeprazine, loxapine, mesoridazine, perphenazine, pimozide, pipotiazine, tetrabenazine, thiopropazate, thioproperazine, thiproperazine, thioridazine, thiothixene, trifluoperazine, and zuclopenthixol). The authors also conducted a secondary nested-case-control analysis, but data are not presented in detail and are therefore not included in this review. ^2^ Polcwiartek et al. (2017) [39] presented three adjusted models in their nested case-control analysis: Model 1: Charlson Comorbidity Index (CCI): disregarded diagnoses included in the CCI < 1 year prior to event. Low, CCIH score 1, moderate/high scores, CCI score ≥ 2; Model 2: Diabetogenic co-medication exposure (within 3 months prior to event): thiazides, beta blockers, oral contraceptives, glucocorticoids, and calcineurin inhibitors; Model 3: psychotropic co-medication exposure (within 3 months prior to event): antiepileptics, anticholinergics, lithium, benzodiazepines, and antidepressants.

**Table 4:** Characteristics of Descriptive Observational Studies on the association between antipsychotic drugs and diabetic ketoacidosis or hyperosmolar hyperglycemic state ^1,2^.

Author (year)	Exposure/Outcome	Results	
Ely et al. (2013) [43]	**Exposure:** **Atypical APDs ^1^** • Quetiapine (*n* = 7) • Olanzapine (*n* = 6) • Risperidone (*n* = 3) • Ziprasidone (*n* = 1) • Clozapine (*n* = 1) **Typical APD** Chlorpromazine (*n* = 0) **Outcome:** • DKA	**Descriptive** **Sample characteristics: *n*** • Total number of deaths with hyperglycemia and DKA: 17 • Medical history of DM: 1 • Deaths with evidence of psychiatric therapy or history: **Psychiatric condition: *n*** • SCZ: 10 • SCZ/BD: 3 • BD: 2 • Major depression: 2 **Metabolic markers** • Mean glucose—vitreous or urine—(mg/dL): 1095 (range: 417–4256) • Blood ketones: ***n* =** 17	
Galler et al. (2015) [44]	**Exposure:** **Among subjects with T1DM aged < 25 years (*n* = 291)** **Atypical APD: *n*; %** • Risperidone: 122; 42 • Quetiapine: 19; 7 • Sulpiride: 10; 3 • Olanzapine: 9; 3 • Others: 11; 4 **Typical APD, (*n* =; %)** • Pipamperone: 29; 10 • Promethazine: 18; 6 • Prochlorperazine: 17; 6 • Haloperidol: 16; 6 • Chlroprothixene: 12; 4 • Pimozide: 10; 3 • Levomepromzaine: 9; 3 • Others: 24; 8 • Concomitant use of atypical and typical APDs: 15; 5 **Outcome:** • Rate of episodes of DKA (per 1 patient-year)	**Descriptive** **Sample characteristics: *n*** • Patients with T1DM < 25 years administered an APD: 291; 0.48% **HbA1c (Median %; Q1–Q3%)** *Without APD With APD* 7.9 (7.1, 9.2) 8.2 (7.3, 9.6) (*p* = 0.008) (Kruskal-Wallis test) **Rate of episodes of DKA (Rate per 1 patient-year (CI range))** *Without APD With APD* 0.06 (0.001) 0.16 (0.03) *p* < 0.0001 (Poisson Model)	**Analytical** **Linear regression analysis (adjusted for age, sex, and DM duration, DM center):** **HbA1c (%; mmol/mol)** **Atypical:** Without APD With APD 8.4; 68 8.7; 72 (*p* = 0.022) **Typical APD:** Without APD With APD 8.4; 68 8.2; 66 (*p* = 0.15) **Any APD:** Without APD With APD 8.4; 68 8.4; 68 (*p* = 0.45) **Poisson regression analysis (adjusted for age, sex, and DM duration, DM center):** **Rates of episodes of DKA events (per 1 patient-year)** **Atypical APD:** Without APD With APD 0.05 0.12 (*p* < 0.001) **Typical APD:** Without APD With APD 0.05 0.13 (*p* < 0.001) **Any APD:** Without APD With APD 0.05 0.12 (*p* < 0.001)
Jin et al. (2002) [45]	**Exposure:** Atypical APDs • Clozapine • Olanzapine • Quetiapine • Risperidone **Outcome:** DKA	**Descriptive** **Sample characteristics:** • Cases with new DM or DKA: ***n =*** 45 DKA cases: *n*; % • DKA: 19; 42 • No DKA: 26; 58 • APDs for DM and DKA (not stratified): *n*; % ▪ Clozapine 20; 44 ▪ Olanzapine 19; 42 ▪ Risperidone *n* = 3; 7 ▪ Quetiapine *n* = 3; 7 • New onset DM presenting as DKA: 42% • BG at presentation > 500 mg/dL **Comparison of patient characteristics for those who developed DM only vs. DKA associated with the use of atypical APDs:** Mean BG mg/dL ± SD; *n* • DM only 481 ± 360; 26 • DKA 750 ± 396; 19 (*t*-test = 2.34; *p* = 0.024)	
Koller et al. (2001; 2003; 2004) Koller & Doraiswamy (2002) ^1^ [46,47,48,49]	**Exposure:** Atypical APD: • Clozapine [45] • Olanzapine [46] • Risperidone [47] • Quetiapine [48] Atypical APD: Haloperidol [47] **Outcome:** • DKA • Acidosis • Ketosis • HHS	**Descriptive**	
		**Koller (2001)-Clozapine** [46], ***n*** • Metabolic acidosis or ketosis (not differentiated): 80 ▪ BG levels ≥ 700 mg/dL: 26 ▪ Occurrence within 3 months of APD initiation: 32; 63% ▪ New onset DM: 73; 91% • Deaths with ketosis or acidosis: 16 ▪ BG levels ≥ 700 mg/dL: 6 ▪ Post-mortem euglycemic DKA: 1 **Koller (2002)-Olanzapine** [47] • Metabolic acidosis or ketosis (not differentiated): 80 ▪ BG ≥ 700 mg/dL: 41 ▪ New-onset DM: 74; 92% • Deaths with metabolic acidosis/ketosis: 10 ▪ Shortly after hyperglycemic event: 9 ▪ 8 months after APD initiation:1 • Recurrence of DKA after reinstitution of olanzapine: 1 • HHS: 2 ▪ BG = 1676 mg/dL and 800 mg/dL **Addendum (post-publication)** • Hyperglycemia or new DM: 52 ▪ Ketosis or acidosis: 20 ▪ BG ≥ 700 mg/dL: 10 ▪ HHS: 1	**Koller 2003-Risperidone** [48], ***n*** Ketoacidosis or ketosis, not differentiated **•** In the presence of hyperglycemia: 26 ▪ Risperidone MT: 24 ∘ New onset DM: 22 ∘ Exacerbation of pre-existing DM: 3 ∘ Unspecified hyperglycemia: 1 ▪ Concomitant use (risperidone & haloperidol): *n* = 2 ∘ New-onset DM: 1 ∘ Exacerbation of pre-existing DM: 1 • Deaths with ketosis or acidosis *n* = 3 **Koller 2004-Quetiapine** [49], ***n*** Hyperglycemia or new DM: 46 Acidosis or ketosis, not differentiated: 21 ▪ BG ≥ 500 mg/dL: 19 ▪ Re-challenge in pre-existing DM: 1 ▪ Deaths: 11; 7 with acidosis or ketosis **Addendum (post-publication):** **•** Hyperglycemia or new DM: 23 ▪ DKA: 4 ▪ Metabolic acidosis associated with Ketosis: 7 (renal failure-related case excluded from this summary) ▪ HHS: 1

Abbreviations: APD, Antipsychotic Drug; BD, Bipolar Disorder; DM, Diabetes Mellitus; HHS, hyperglycemic hyperosmolar state; Q, Quartile; SCZ, Schizophrenia; SD, Standard Deviation; T1DM, Type 1 Diabetes Mellitus; *t*-test, Student’s *t*-test; > greater than; ≥, greater than or equal to; < less than. ^1^ Koller et al. (2001; 2002; 2003; 2004) [46,47,48,49] analyzed different hyperglycemic complications, including DKA and/or HHS. The authors specified that metabolic abnormalities ranged from mild glucose intolerances to DKA and HHS. The studies did not always differentiate between all the hyperglycemic complications, and demographic information is also presented for acidosis and ketosis. Cause of death in these analyses was not always ascertained to be due to ketosis or acidosis. ^2^ The analysis by Suzuki et al. (2013) [35] concluded there was no serious adverse DKA events occurred between the two groups administered: intramuscular olanzapine and intramuscular haloperidol. Therefore, it is not included in this Table.

## Data Availability

No new data were generated or analyzed in this study. All data used were obtained from publicly available studies.

## Supplementary Materials

The following supporting information can be downloaded at https://www.mdpi.com/article/10.3390/jcm15093536/s1, Supplemental Material I: Preferred Reporting Item for Systematic Reviews and Meta-Analyses Extension for Scoping Reviews (PRISM-ScR) Checklist (Table S1); Supplemental Material II: Search Strategy; Supplemental Material III: Distiller SR form for level 1 screening; Supplemental Material IV: Additional Detailed Information for included Observational Studies (Tables S2, S3 and S4).

## Abbreviations

The following abbreviations are used in this manuscript:

AE	Adverse Event
ADR	Adverse Drug Report
5-HT2	5-hydroxytryptamine type 2 receptor
APD	Antipsychotic Drug
BD	Bipolar Disorder
CI	Confidence Intervals
D2	Dopamine 2
DM	Diabetes Mellitus
DKA	Diabetic Ketoacidosis
FAERS	Food and Drug Adverse Reporting System
FDA	Food and Drug Administration
FGA	First Generation Antipsychotic
HR	Hazard Ratio
HbA1c	Hemoglobin A1c
HHS	Hyperglycemic Hyperosmolar State
JARED	Japanese Adverse Drug Event Report Database
MT	Monotherapy
PRR	Proportional Reporting Ratio
PT	Preferred Term
ROR	Reporting Odds Ratio
SOC	System Organ Classification
T1DM	Type 1 Diabetes Mellitus
T2DM	Type 2 Diabetes Mellitus
SCZ	Schizophrenia
SD	Standard Deviation
SGA	Second Generation Antipsychotic
SZA	Schizoaffective Disorder
