# Supplementary material for: Antipsychotic Drugs and the Risk of Diabetic Complications: A Systematic Review of Observational Studies
Source: J Clin Med. 2026 May 6;15(9):3536. doi: 10.3390/jcm15093536 (PMC13163739; doi:10.3390/jcm15093536)
Supplement: Supplementary file 1 [file jcm-15-03536-s001.zip › jcm-4156397-supplementary.pdf]

Antipsychotic Drugs and the Risk of Diabetic Complications: A systematic Review of Observational Studies

Nisrine Haddad [1,*], Nawal Farhat [1,2], Christopher Gravel [1,3,4], Yue chen [1], Franco Momoli [1,5], Donald Mattison [1,5,6], Jeannette Guogen [7,8], Daniel Krewski [1,2,5]

[1] School of Epidemiology and Public Health, University of Ottawa, Ottawa, ON, Canada
[2] School of Mathematics and Statistics, Carleton University, Ottawa, ON K1S 5B6, Canada
[3] Department of Mathematics and Statistics, University of Ottawa, Ottawa, ON K1N 6N5, Canada
[4] Data Literacy Research Institute, University of Ottawa, Ottawa, ON K1N 6N5, Canada
[5] Risk Sciences International, Ottawa, ON, K1Z 7T1, Canada
[6] Arnold School of Public Health, University of South Carolina, Columbia, South Carolina, United States
[7] Department of Medicine, University of Toronto, ON M5S 3H2, Canada
[8] Division of Endocrinology, St. Michael's Hospital, ON M5C 2T2, Canada
* Correspondence: nhadd026@uottawa.ca

SUPPLEMENTAL MATERIAL

The following supplemental material provides the PRISMA checklist (Supplemental Material I) search strategy using four bibliographic databases (Supplemental Material II), the Distiller SR form for level 1 screening (Supplemental Material III). Supplemental Material IV contains additional detailed information for each observational study are presented (Tables S2 and S3) and the quality assessment and risk of bias ratings for included studies (Table S4).

Supplemental Material I: PRISMA Checklist

Table S1. Preferred Reporting Items for Systematic reviews and Meta-Analyses extension for Scoping Reviews (PRISMA-ScR) Checklist.

SECTION	ITEM	PRISMA-ScR CHECKLIST ITEM	REPORTED ON PAGE #
**TITLE**			
Title	1	Antipsychotic Drugs and the Risk of Diabetic Complications: A Systematic Review of Observational Studies	1
**ABSTRACT**			
Structured summary	2	Provide a structured summary that includes (as applicable): background, objectives, eligibility criteria, sources of evidence, charting methods, results, and conclusions	1

SECTION	ITEM	PRISMA-ScR CHECKLIST ITEM	REPORTED ON PAGE #
		that relate to the review questions and objectives.	
**INTRODUCTION**			
Rationale	3	Describe the rationale for the review in the context of what is already known. Explain why the review questions/objectives lend themselves to a scoping review approach.	2,3
Objectives	4	Provide an explicit statement of the questions and objectives being addressed with reference to their key elements (e.g., population or participants, concepts, and context) or other relevant key elements used to conceptualize the review questions and/or objectives.	3
**METHODS**			
Protocol and registration	5	Indicate whether a review protocol exists; state if and where it can be accessed (e.g., a Web address); and if available, provide registration information, including the registration number.	N/A
Eligibility criteria	6	Specify characteristics of the sources of evidence used as eligibility criteria (e.g., years considered, language, and publication status), and provide a rationale.	5
Information sources*	7	Describe all information sources in the search (e.g., databases with dates of coverage and contact with authors to identify additional sources), as well as the date the most recent search was executed.	3,4
Search	8	Present the full electronic search strategy for at least 1 database, including any limits used, such that it could be repeated.	Supplemental Material II
Selection of sources of evidence†	9	State the process for selecting sources of evidence (i.e., screening and eligibility) included in the scoping review.	5,6
Data charting process‡	10	Describe the methods of charting data from the included sources of evidence (e.g., calibrated forms or forms that have been tested by the team before their use, and whether data charting was done	4-6

SECTION	ITEM	PRISMA-ScR CHECKLIST ITEM	REPORTED ON PAGE #
		independently or in duplicate) and any processes for obtaining and confirming data from investigators.	
Data items	11	List and define all variables for which data were sought and any assumptions and simplifications made.	4,5
Critical appraisal of individual sources of evidence§	12	If done, provide a rationale for conducting a critical appraisal of included sources of evidence; describe the methods used and how this information was used in any data synthesis (if appropriate).	5,6
Synthesis of results	13	Describe the methods of handling and summarizing the data that were charted.	4-6
**RESULTS**			
Selection of sources of evidence	14	Give numbers of sources of evidence screened, assessed for eligibility, and included in the review, with reasons for exclusions at each stage, ideally using a flow diagram.	3-5
Characteristics of sources of evidence	15	For each source of evidence, present characteristics for which data were charted and provide the citations.	4-6 Tables 2-4 Supplemental Material: Tables S2, S3
Critical appraisal within sources of evidence	16	If done, present data on critical appraisal of included sources of evidence (see item 12).	5,6 Supplemental Material: Table S4
Results of individual sources of evidence	17	For each included source of evidence, present the relevant data that were charted that relate to the review questions and objectives.	Tables 3 and 4 Supplemental Material: Tables S2 and S3
Synthesis of results	18	Summarize and/or present the charting results as they relate to the review questions and objectives.	6-18
**DISCUSSION**			
Summary of evidence	19	Summarize the main results (including an overview of concepts, themes, and types of evidence available), link to the review	18-21

SECTION	ITEM	PRISMA-ScR CHECKLIST ITEM	REPORTED ON PAGE #
		questions and objectives, and consider the relevance to key groups.	
Limitations	20	Discuss the limitations of the scoping review process.	22
Conclusions	21	Provide a general interpretation of the results with respect to the review questions and objectives, as well as potential implications and/or next steps.	22
**FUNDING**			
Funding	22	Describe sources of funding for the included sources of evidence, as well as sources of funding for the scoping review. Describe the role of the funders of the scoping review.	N/A

Supplemental Material II: Search Strategies-Updated up to 17 October 2025

MEDLINE

1 Aripiprazole/ 3154

2 (abilify or Aripiprazole or asenapine maleate or saphris or clozaril or clozapine or iloperidone or fanapt or lurasidone or latuda or olanzapine* or zyprexa or symbyax or paliperidone or invega or quetiapine or seroquel or risperidone or risperdal or ziprasidone or geodon or cariprazine or Vraylar or Pimavanserin or Nuplazid).ti,ab,kw. 37361

3 clozapine/ 9937

4 Lurasidone Hydrochloride/ 435

5 Paliperidone Palmitate/ 1139

6 Quetiapine Fumarate/3354

7 Risperidone/ 7056

8 Pimavanserin/0

9 cariprazine/ 0

10 1 or 2 or 3 or 4 or 5 or 6 or 7 or 8 or 9 39801

11 diabetes complications/ or diabetic coma/ or hyperglycemic hyperosmolar nonketotic coma/ or diabetic ketoacidosis/ 55044

12 (diabet* adj3 complication*).ti,ab,kw.43885

13 hyperglycemi*.ti,ab,kw. 70426

14 (diabet* adj3 ketoacidosis).ti,ab,kw. 9401

15 (diabet* adj3 (coma* or hyperglycemi*)).ti,ab,kw. 10002

16 11 or 12 or 13 or 14 or 15 158292

17 10 and 16 398

18 HALOPERIDOL/ 16274

19 (Haloperidol or Haldol or Loxapine or Loxitane or Molindone or Moban or Thiothixene or Navane or Primozide or Orap or Trifluoperazine or Stelazine or Chlorpromazine or

Thorazine or Fluphenazine or Perphenazine or "Perphenazine amitriptyline" or Prochlorperazine or Thioridazine or Droperidol or Inapsine).ti,ab,kw. 43221

20 CHLORPROMAZINE/ 17477

21 FLUPHENAZINE/ 2441

22 Loxapine/ 327

23 PERPHENAZINE/ 1599

24 Prochlorperazine/ 1101

25 PROCHLORPERAZINE/ 1101

26 THIORIDAZINE/ 2406

27 TRIFLUOPERAZINE/3636

28 THIOTHIXENE/ 335

29 Thioridazine/ 2406

30 Droperidol/ 2036

31 LOXAPINE/ 327

32 MOLINDONE/ 145

33 PIMOZIDE/ 1757

34 Fluphenazine/ 2441

35 18 or 19 or 20 or 21 or 22 or 23 or 24 or 25 or 26 or 27 or 28 or 29 or 30 or 31 or 32 or 33 or 34 58621

36 16 and 35 159

37 17 or 36 515

38 10 or 35 91913

39 16 and 38 515

40 limit 39 to ed=20200901-20251015 62

41 limit 39 to dt=20200901-20251015 83

42 40 or 41 91

CENTRAL

1 Aripiprazole/ 743

2 (abilify or Aripiprazole or asenapine maleate or saphris or clozaril or clozapine or iloperidone or fanapt or lurasidone or latuda or olanzapine* or zyprexa or symbyax or paliperidone or invega or quetiapine or seroquel or risperidone or risperdal or ziprasidone or geodon or cariprazine or Vraylar or Pimavanserin or Nuplazid).ti,ab,kw. 10907

3 clozapine/ 606

4 Lurasidone Hydrochloride/ 153

5 Paliperidone Palmitate/ 316

6 Quetiapine Fumarate/849

7 Risperidone/ 1614

8 Pimavanserin/9

9 cariprazine/ 6

10 1 or 2 or 3 or 4 or 5 or 6 or 7 or 8 or 9 11121

11 diabetes complications/ or diabetic coma/ or hyperglycemic hyperosmolar nonketotic coma/ or diabetic ketoacidosis/ 1802

12 (diabet* adj3 complication*).ti,ab,kw.5739

13 hyperglycemi*.ti,ab,kw. 8890

14 (diabet* adj3 ketoacidosis).ti,ab,kw. 992

15 (diabet* adj3 (coma* or hyperglycemi*)).ti,ab,kw. 663

16 11 or 12 or 13 or 14 or 15 16261

17 10 and 16 68

18 HALOPERIDOL/ 1576

19 (Haloperidol or Haldol or Loxapine or Loxitane or Molindone or Moban or Thiothixene or Navane or Primozide or Orap or Trifluoperazine or Stelazine or Chlorpromazine or Thorazine or Fluphenazine or Perphenazine or "Perphenazine amitriptyline" or Prochlorperazine or Thioridazine or Droperidol or Inapsine).ti,ab,kw. 6001

20 CHLORPROMAZINE/ 653

21 FLUPHENAZINE/ 302

22 Loxapine/ 80

23 PERPHENAZINE/ 215

24 Prochlorperazine/ 216

25 PROCHLORPERAZINE/ 216

26 THIORIDAZINE/ 200

27 TRIFLUOPERAZINE/122

28 THIOTHIXENE/ 74

29 Thioridazine/ 200

30 Droperidol/ 522

31 LOXAPINE/ 80

32 MOLINDONE/ 23

33 PIMOZIDE/ 123

34 Fluphenazine/ 302

35 18 or 19 or 20 or 21 or 22 or 23 or 24 or 25 or 26 or 27 or 28 or 29 or 30 or 31 or 32 or 33 or 34 6681

36 16 and 35 15

37 17 or 36 76

38 10 or 35 16110

39 16 and 38 76

40 limit 39 to yr="2020-current" 18

EMBASE

1 Aripiprazole/ 23479

2 (abilify or Aripiprazole or asenapine maleate or saphris or clozaril or clozapine or iloperidone or fanapt or lurasidone or latuda or olanzapine* or zyprexa or symbyax or paliperidone or invega or quetiapine or seroquel or risperidone or risperdal or ziprasidone or geodon or cariprazine or Vraylar or Pimavanserin or Nuplazid).ti,ab,kw. 61127

3 clozapine/ 40526

4 Lurasidone Hydrochloride/ 3131

5 Paliperidone Palmitate/ 7448

6 Quetiapine Fumarate/32505

7 Risperidone/ 47067

8 Pimavanserin/1077

9 cariprazine/ 1478

10 1 or 2 or 3 or 4 or 5 or 6 or 7 or 8 or 9 107324

11 diabetes complications/ or diabetic coma/ or hyperglycemic hyperosmolar nonketotic coma/ or diabetic ketoacidosis/ 34756

12 (diabet* adj3 complication*).ti,ab,kw.65822

13 hyperglycemi*.ti,ab,kw. 107838

14 (diabet* adj3 ketoacidosis).ti,ab,kw. 16083

15 (diabet* adj3 (coma* or hyperglycemi*)).ti,ab,kw. 14086

16 11 or 12 or 13 or 14 or 15 189539

17 10 and 16 1074

18 HALOPERIDOL/ 64903

19 (Haloperidol or Haldol or Loxapine or Loxitane or Molindone or Moban or Thiothixene or Navane or Primozide or Orap or Trifluoperazine or Stelazine or Chlorpromazine or Thorazine or Fluphenazine or Perphenazine or "Perphenazine amitriptyline" or Prochlorperazine or Thioridazine or Droperidol or Inapsine).ti,ab,kw. 47553

20 CHLORPROMAZINE/ 43669

21 FLUPHENAZINE/ 10115

22 Loxapine/ 2864

23 PERPHENAZINE/ 7577

24 Prochlorperazine/ 6720

25 PROCHLORPERAZINE/ 6720

26 THIORIDAZINE/ 12383

27 TRIFLUOPERAZINE/10351

28 THIOTHIXENE/ 2663

29 Thioridazine/ 12383

30 Droperidol/ 10025

31 LOXAPINE/ 2864

32 MOLINDONE/ 1300

33 PIMOZIDE/ 8699

34 Fluphenazine/ 10115

35 18 or 19 or 20 or 21 or 22 or 23 or 24 or 25 or 26 or 27 or 28 or 29 or 30 or 31 or 32 or 33 or 34 129700

36 16 and 35 719

37 17 or 36 1447

38 10 or 35 204295

39 16 and 38 1447

40 limit 39 to yr="2020 -Current" 354

41 limit 39 to dc=20200901-20251015 380

42 limit 39 to dd=20200901-20251015 381

43 41 or 42 383

PsycINFO

1 Aripiprazole/ 2079

2 (abilify or Aripiprazole or asenapine maleate or saphris or clozaril or clozapine or iloperidone or fanapt or lurasidone or latuda or olanzapine* or zyprexa or symbyax or paliperidone or invega or quetiapine or seroquel or risperidone or risperdal or ziprasidone or geodon or cariprazine or Vraylar or Pimavanserin or Nuplazid).ti,ab,mp. 24625

3 clozapine/ 5847

4 Lurasidone Hydrochloride/ 0

5 Paliperidone Palmitate/ 0

6 Quetiapine Fumarate/0

7 Risperidone/ 4102

8 Pimavanserin/0

9 cariprazine/ 0

10 1 or 2 or 3 or 4 or 5 or 6 or 7 or 8 or 9 24625

11 diabetes complications/ or diabetic coma/ or hyperglycemic hyperosmolar nonketotic coma/ or diabetic ketoacidosis/ 0

12 (diabet* adj3 complication*).ti,ab,mp. 2219

13 hyperglycemi*.ti,ab,mp. 2132

14 (diabet* adj3 ketoacidosis).ti,ab,mp. 210

15 (diabet* adj3 (coma* or hyperglycemi*)).ti,ab,mp. 208

16 11 or 12 or 13 or 14 or 15 4359

17 10 and 16 231

18 HALOPERIDOL/ 4868

19 (Haloperidol or Haldol or Loxapine or Loxitane or Molindone or Moban or Thiothixene or Navane or Primozide or Orap or Trifluoperazine or Stelazine or Chlorpromazine or Thorazine or Fluphenazine or Perphenazine or "Perphenazine amitriptyline" or Prochlorperazine or Thioridazine or Droperidol or Inapsine).ti,ab,mp. 16702

20 CHLORPROMAZINE/ 1763

21 FLUPHENAZINE/ 550

22 Loxapine/ 93

23 PERPHENAZINE/ 199

24 Prochlorperazine/ 27

25 PROCHLORPERAZINE/ 27

26 THIORIDAZINE/ 373

27 TRIFLUOPERAZINE/146

28 THIOTHIXENE/ 104

29 Thioridazine/ 373

30 Droperidol/ 0

31 LOXAPINE/ 93

32 MOLINDONE/ 48

33 PIMOZIDE/ 491

34 Fluphenazine/ 550

35 18 or 19 or 20 or 21 or 22 or 23 or 24 or 25 or 26 or 27 or 28 or 29 or 30 or 31 or 32 or 33 or 34 17065

36 16 and 35 53

37 17 or 36 250

38	10 or 35	37280
39	16 and 38	250
40	limit 39 to up=20200901-20251015	19

Supplementary Material III: Level 1-Title and Abstract Screening

1. Should this study be included in Stage 2 screening?

- ◯ Yes
- ◯ Unsure
- ◯ No

2. Reason for exclusion:

- ☐ Population
- ☐ Interventions(s)/Exposure(s)
- ☐ Comparison
- ☐ Outcome(s)
- ☐ Study Design

Supplemental Material IV-Detailed Description of Included Studies (Tables S2 and S3), with and Quality and Risk of Bias Assessment (Table S4).

Table S2. Characteristics of Analytical Observational Studies[1].

1st author (publication year) Inclusion/ Exclusion Criteria	Brief Description of Statistical Methods	Age of Study Participants (years)	Sex of Study Participants	Ethnicity of Study Participants	Strengths /Limitations
**Henderson et al. (2007) [36]**<br>**Inclusion:**<br>•Patients diagnosed with schizophrenic disorders (ICD-9 295.0-295.3)<br>•DKA and HHS (250.1-250.3)<br>•Record of an atypical APD: clozapine, olanzapine, risperidone, quetiapine and ziprasidone. **Exclusion:**<br>•24 cases did not meet ICD-9 or DSM-IV criteria for diagnosis of SCZ, SZA, DKA or HHS; •1 patient eliminated because of steroid-induced HHS	•The total person years was calculated as a cumulative follow-up time per patient over the study period.<br>•For patients presenting with SCZ or SZA, the total duration of exposure to an atypical APD was calculated for each person.<br>•The total number of patients receiving treatment with each atypical APD over the 7-year study period were recorded.<br>•For DKA/HHS cases only the time preceding the event was included..<br>•Incidence of DKA or HHS: total events / by total person-years per 10,000 patient years.<br>•Comparison with the general population: ▪Used the 1.4/10,000 patient years of DM presenting as DKA as a reference.<br>▪Group comparisons performed using X^2 test, t-tests of DM presenting as DKA and DKA in known DM patients.<br>▪Statistical significance: $p < 0.05$.	**New-onset DM:**<br>DKA (*n* = 11):<br>mean ± SD: 42 ± 12 years (range: 26-64)<br><br>**New-onset DKA in patients with DM** (*n* = 8):<br>mean ± SD: 44 ± 12 years (range: 23-64)<br><br>HHS:<br>•Case 1,	**For the entire sample:**<br>•Male (*n* = 401,461, 49%)<br>•Female (*n* = 417,847, 51%)<br><br>**Patients diagnosed with SCZ or SD** (n = 4,850):<br>•Male (*n* = 2,619, 54%)<br>•Female (*n* = 2,231, 46%)<br><br>**Patients with new onset DM presenting with DKA:**<br>•Male (*n* = 5, 45%)	**Patients diagnosed with SCZ or SZA** (n = 4,850):<br>•White, *n* = 3,584 (74%)<br>•Black, *n* = 631 (13%)<br>•Hispanic, *n* = 326 (7%)<br>•Asian, *n* = 69 (1%)<br><br>**New onset DM presenting as DKA** (*n* = 11)<br>•White, *n* = 8 (73%)<br>•Black, *n* = 2 (18%)<br>•Hispanic, *n* = 1 (9%)	**Strengths:**<br>•Results are consistent with studies in the non-psychiatric general population.<br>**Limitations:**<br>•Underestimation of the DKA events: Patients may have received treatment for DKA at different institutions and records are not available.<br>•Patients were treated with an APD that was not recorded in inpatient/outpatient records.<br>•Residual confounding: Prior treatment with APDs was not controlled for.

•3 patients eliminated because of chronic pancreatitis or cirrhosis)		age= 55<br>•Case 2, age = 29	•Female (*n* = 6, 55%)	Known DM presenting as DKA (*n* = 8)<br><br>•White, *n* = 5 (63%)<br>•Black, *n* = 2 (25%)<br>•Hispanic, *n* = 1 (12%)	
**Leslie & Rosenheck (2004) [37]**<br>**Inclusion:**<br>•Patients consistently prescribed APD monotherapy for 3-months.<br>•Patients without a DM diagnosis.<br>**Exclusion:**<br>•Patients treated with ziprasidone or aripiprazole.<br>•Patients with an outpatient claims for DM (*n* = 11,069) or < 2 medical primary care visits (*n* = 6,028) in the previous 6 months.	•Cox proportional hazards models used to estimate time to DKA hospitalization.<br>•Adjustments: APDs prescribed during the stable period, end date of the stable period, demographic variables (age, gender, race), income, comorbid mental health diagnoses, levels of service use during the stable period, degree of VA service-connected disability.<br>•Attributable risk estimated for atypical APD vs conventional APDs	NR	NR	NR	**Strengths:**<br>• Accrued knowledge into the risk DKA in an older, male population with schizophrenia for whom APD MT is prescribed.<br>**Limitations:**<br>•Findings may not be generalizable to other populations or health care systems.<br>•Unidentified cases of DKA outside the Department of Veterinary Affairs<br>•Missing data: Medications prescribed before/after the APD identified for the study may have contributed to the risk of DKA.

**Lipscombe et al. (2014)** [1] **[38]**<br>**Inclusion:**<br>•Patients ≥ 18 years and newly treated with an APD (no prescription in the preceding 365 days prior to cohort entry). .<br>**Exclusion:**<br>•Patient < 18 years of age on the cohort entry<br>•66 years of age in the provinces where drug benefit coverage started at age 65 (Nova Scotia, Ontario and Alberta)<br>•Less than 365 days of enrollment in their health plan<br>•Resided in a nursing home<br>• Received more than one APD<br>•Hospitalized with the primary study outcome within 30 days preceding cohort entry<br>•Hospitalized for ≥ 30 consecutive days in the year preceding cohort entry	• One primary and two secondary pair-wise comparisons of the risk of a hyperglycemic emergency<br>►Reference group: Risperidone<br>►Primary comparator: Olanzapine<br>► Secondary comparators: Other typical and atypical antipsychotics<br>• Propensity score with inverse probability of treatment weighting (IPTW) to estimate marginal treatment effects.<br>• Regression on demographic variables (age, sex), other variables: calendar year of cohort entry; history of diabetes, schizophrenia, and dementia, neighbourhood income quintile, Romano Comorbidity Index; number of hospitalizations, outpatient family physician consultations and outpatient psychiatric consultations in the year preceding cohort entry; and concurrent exposure to other medication<br>•The CPRD team added other variables: smoking status, alcohol use, and BMI.<br>•Absolute difference in the probability of a hyperglycemic emergency within 365 days of drug initiation was estimated.<br>•<br>•Estimation of the cause-specific hazard: subjects were censored on the occurrence of death.<br>•Treatment effects were stratified by age group: 18–65 and ≥ 66, and by pre-existing DM status.<br>•Site-level analysis completed independently and results were blinded. •Meta-analyses were performed by a team member not involved in the site-level analyses using fixed-effect models with inverse variance weighting.<br>• Heterogeneity was assessed using I^2.<br>•Sensitivity analysis: using random-effects models	•18-65 (45%)<br>• ≥ 66 years (55%)	18 – 65 years:<br>• Male (*n* = 154,095; 47.5%)<br>• Female (*n* = 170,417; 52.5%)<br>≥ 66 years:<br>• Male (*n* = 158,132; 39.4%)<br>• Female (*n* = 242,845; 60.6%)	NR	**Strengths:**<br>•Analysis limited to new users of APDs with similar indication of use.<br>•Outcome was hospital visits for hyperglycemic emergencies which are consistently coded.<br>•Adjusted for treatment characteristics at baseline (e.g., diabetes severity, dementia).<br>**Limitations:**<br>•Selection bias due to confounding by indication.<br>•Residual confounding (e.g., medication adherence)

•Received renal dialysis or palliative care in the year preceding cohort entry.					
**Polcwiartek et al. (2017) [39]**<br>**Inclusion:**<br>•Patients diagnosed schizophrenia (ICD-10: F20.x) in the Danish Psychiatric Central Research Register.<br>•Cases were defined as having an incident onset of DKA event identified using ICD-10 discharge diagnoses: E1x.1, E10.0B, E11.0E or E12.0C.<br>Controls: Risk-set sampling method matched by age, sex and year of schizophrenia onset<br>•5 randomly selected controls who were alive and had not migrated prior to DKA.<br>**Exclusion:**<br>**Cases and controls:**<br>•Diagnosis from	•Continuous variables: non-parametric Mann-Whitney U test<br>•Categorical variables: Fisher's exact or the X^2 test<br>•Controls were assigned an index date identical to that of the corresponding case.<br>•Conditional logistic regression: to estimate the association between APD exposure and DKA<br>•Crude and adjusted ORs (95% CIs) with no APD exposure as a reference.<br>•Age and sex were accounted for through the matching procedure.<br>•Adjustments for confounders:<br>1. Charlson Comorbidity Index (CCI): disregarded diagnoses included int he CCI < 1 year prior to event. Low, CCIH score 1, moderate/High scores, CCI score ≥ 2.<br>2. Diabetogenic co-medication exposure (within 3 months prior to event): thiazides, beta blockers, oral contraceptives, glucocorticoids, calcineurin inhibitors<br>3. psychotropic co-medication exposure (within 3 months prior to event): antiepileptics, anticholinergics, lithium, benzodiazepines, antidepressants.<br>•Co-medication exposure: included as a count-basedvariable reflecting increasing number of concurrent medications and each additional medication (was assumed to increase the odds ration proportionally)<br>• Data management and analysis were performed using	•Adults > 18 years<br>**DKA cases** (*n* = 28)<br>**Control cases** (*n* = 137)<br><br>Median, IQR<br>•Age at first APD exposure in years:<br>Cases: 32.4 (26.6-37.1)<br>Controls: 30.8 (26.2-35.8)<br><br>•Age at schizophrenia onset in years: Cases: 32.3 (27.1 -37.6)<br>Controls: 32.5 (27.7 -37.6)<br>•Age at DKA	•Cases:<br>▪21 males (75%)<br>▪7 females (25%)<br><br>Controls<br>▪102 males (74.5%)<br>▪35 females (25.5%)	NR	**Strengths:**<br>•Use of nationwide Danish healthcare registers.<br>**Limitations:**<br>•Observational design: causal inference cannot be established.<br>•Bias: Unmeasured/Unknown confounders due to unavailability of variables such as: family history of diabetes, ethnicity, lifestyle, BMI, severity of SCZ, medication adherence, and changes in prescription patterns.<br>•Confounding by indication may underestimate the risks of DKA associated with APDs<br>•Unable to distinguish DKA from other metabolic emergencies such as lactic acidosis and HHS.<br>•Due to low numbers, unable to evaluate exposure

emergency room visits due to low validity.<br>•With prior history of pancreatitis, pancreatic cancer or polycystic ovary syndrome.	Stata, version 14.1 and R software, version 3.3.2<br>● All analysis were two-sided.<br>•Statistical significance: $p < 0.05$	onset/index date in years:<br>Cases: 37.7 (30.7-44.6)<br>Controls: 36.8 (29.5-44.2)			to single APDs<br>•Cumulative carry-over exposure effects although analysis included APD exposure within 3 months prior to DKA occurrence.<br>•Use of non-exposed reference group may introduce heterogeneity in health status and access to care.
**Ramaswamy et al. (2007) [40]**<br>**Inclusions:**<br>•Patients receiving an atypical APD<br>•Initial APDprescription: first prescription for an antipsychotic agent after a 6-month period during which there were no claims for antipsychotic medications.<br>Cases:<br>[ICD-9] code 2501x) after initial prescription of an APD and one atypical APD 45 days	•Logistic regression: RR of DKA<br>•Adjustment for potential confounding factors: Treatment groups: olanzapine or risperidone; demographic variables: age, sex and race) months of Medicaid eligibility; history of DM prior to APD use; mental health diagnosis; dispensation of a conventional APD or diabetogenic agent within 45 days of DKA event).<br>• Interactions were tested between treatment group and predictors that different significantly between the two APDs.<br>• Model fit was assessed using the Hosmer and Lameshow test.<br>•Multicollinearity was assessed using variance inflation factors.<br>• All statistical tests were two-tailed.<br>• Analyses were conducted using SAS/STAT software, Version 8.	•Olanzapine: Mean age ± SD: 45.5 ± 14.2<br>•Risperidone: Mean age ± SD: 55.0 ± 21.5	Olanzapine (*n* = 55)<br>•Male (*n* = 23, 41.8%)<br>•Female (*n* = 32, 58.2%)<br><br>Risperidone (*n* = 31)<br>•Male (*n* = 9, 29.0%)<br>•Female (*n* = 22, 71.0%)<br><br>•Data on sex was missing for a small number of patients.	Olanzapine:<br>•White: *n* = 22 (40.0%)<br>•African American *n* = 18 (32.7%)<br>•Other 4 (7.3%)<br>•Missing data: *n* = 11 (20.0%)<br>Risperidone:<br>•White: *n* = 16 (51.6%)<br>•African American: *n* = 6 (19.4%)<br>•Other: *n* = 2 (6.5%)<br>•Missing data: *n* = 7 (22.6%)	**Strengths:**<br>•Findings consistent with published CRs, and some studies..<br>**Limitations:**<br>•Inference of causality is limited due to the small number of variables and the retrospective nature of this analysis.<br><br>• •Limit to the generalizability of findings due to lack of comparator group<br>•Bias: Systematic error introduced due to usage of administrative codes.

prior to DKA occurrence.<br>**Exclusions:**<br>•Cases with APD polypharmacy					
**Liang et al. (2025) [41]**<br>•Inclusion: Records where quetiapine was identified as the primary suspect drug<br>•Only DKA cases were included	•ROR and PRR were used to detect ADR signals.<br>If an AE met the following criteria: ROR>1, PRR≥2, ROR's 95% CI>1, χ2≥4 and case number ≥3, they were considered positive.<br>Lower limit of 95% CI of the ROR(ROR25) was used as a standard or judging signal intensity:<br>►Weak signal: 1<ROR025<50;<br>►Moderate signal: 50≤ROR25<1000<br>►Strong signal: ROR025≥1000	Age (years):<br>•< 20: *n* = 66, 2.17%<br>•20-39: *n* = 444, 14.59%<br>•40-59: *n* = 647, 21.24%<br>•≥ 60: 76, 2.50 %	Male: 1,390 (45.63%)<br>Female: 1,563, (51.31%)<br>Null: 93 (3.05)	NR	•**Strengths:**<br>• Large sample size<br>•Usage of real-world data.<br>**Limitations:**<br>•Biases due to underreporting, overreporting and incomplete data.<br>•Cases collected primarily reflect the US and cannot be generalized to other countries.<br>•Co-medication of quetiapine and other drugs were not evaluated and the impacts on the results cannot be interpreted.

**Sugawara et al. (2023) [42]**<br>•Cases with incomplete information on patient sex and age were excluded from the analysis (*n* = 380)<br>•Patients aged <10 years were also excluded, as the onset of schizophrenia in children younger than age 10 is extremely rare (*n* = 26)	•To evaluate the signals of DKA, the reporting odds ratios (RORs) and 95% confidence intervals (CIs) were calculated.<br>•A case–control analysis was conducted using the JADER dataset to identify risk factors associated with high risk APDs.<br>•Descriptive statistics were calculated for demographic and clinical variables.<br>•For categorical variables, chi-square test was used to compare characteristics between groups.<br>•Multivariable regression analysis with a forward selection method was used to calculate aROR to for each APD.<br>•Dependent variable: onset of DKA<br>•Independent variable: age-stratified group, sex, and reporting year<br>•Significance value: $p < 0.05$.<br>•Statistical analyses performed using SPSS software.	**Age for DKA cases** (*n* = 55, %)<br>•10-19 (*n* = 1, 1.8%)<br>•20-29 (*n* = 6, 10.9%)<br>•30-39 (*n* = 17, 30.9%)<br>•40-49 (*n* = 16, 29.1%)<br>•50-59 (*n* = 9, 16.4%)<br>•60-69 (*n* = 2, 3.6%)<br>•70-79 (*n* = 4, 7.3%)<br>•≥80 (*n* = 0, 0.0%)<br>**Age for non DKA cases** (*n* = 7,380)<br>•10-19 (*n* = 172, 2.3%)<br>•20-29 (*n* = 860, 11.7%)<br>•30-39 (*n* = 1515, 19.2%)<br>•40-49 (*n* = 1517, 20.6%)<br>•50-59 (*n* =	**Sex for DKA cases** (*n* = 55)<br>•Male (*n* = 32, 58.2%)<br>•Female (*n* = 23, 41.8%)<br>**Sex for non DKA cases** (*n* = 7,380)<br>•Male (*n* = 3,828, 51.9%)<br>•Female (*n* = 3, 552, 48.1%)	Asian	**Strengths:**<br>•Large sample size: informative resource for detecting rare AEs and long-term risks.• **Limitations:**<br>• Biases due to underreporting, overreporting and incomplete data.<br>•It was not possible to determine the incidence of DKA since not all cases are included in JARED.<br>•Lack of clinical information such as: blood glucose levels, pH, bicarbonate and missing values for information on drug administration.<br>•The severity of the DKA event could not be assessed. It relied on the definition of the ADA diagnostics criteria for this condition.<br>•Outcome determination was dependent on subjective reporting of reporters.<br>•The difference between remission and recovery in the database could not be

	1450, 19.6%)<br>•60-69 (*n* = 1246, 16.9%)<br>•70-79 (*n* = 561, 7.6%)<br>•≥80 (*n* = 159, 2.2%)	determined.

Abbreviations: ADA, American Diabetes Association; APD, Antipsychotic Drug; aROR, Adjusted Reporting Odds Ratio; BMI, Body mass index; CR, Case Report; DKA, Diabetic ketoacidosis; DM, Diabetes Mellitus; SD, Standard Deviation; DSMV-IV, Diagnostic and Statistical Manual of Mental Disorders, Fourth Edition; I, I-squared statistics; ICD, International Classification of Diseases; IQR, Interquartile range; HHS, Hyperglycemic Hyperosmolar State; SCZ, Schizophrenia; SZA, Schizoaffective Disorder; X^2, chi-square. Symbols: > greater; ≥, greater than or equal to; < less; ≤, less than or equal to. [1]Lipscombe et al. (2014) conducted a secondary nested-case-control analysis, but data are not presented in detail and are therefore not included in this Table.

Table S3. Characteristics of Descriptive Observational Studies[1,2]

1st author (publication year) Inclusion/ Exclusion Criteria	Brief Description of Statistical Methods	Age of Study Participants	Sex of Study Participants	Ethnicity of Study Participants	Strengths /Limitations
**Ely et al. (2013) [43]**<br>**Inclusion**:<br>•Decedents treated with SGA (*n* = 17)<br>**Exclusion:**<br>•Decedents where treatment of SGAs prior to death could not be determined (*n* = 3)<br>►Typical APD: haloperidol (*n* = 1)<br>►Buspirone (*n* = 2)	•Deaths certificates and toxicology data were searched for DKA and hyperglycemia events.<br>•Retrospectively analysed the medical examiner records such as: autopsy, toxicology, police, and medical examiner investigators' reports.<br>•Post-mortem blood was collected and toxicological analysis was completed on all decedents.<br>•Urine or blood were analyzed by gas chromatography for olanzapine, quetiapine, and clozapine.<br>•A reference laboratory analyzed risperidone and ziprasidone.<br>•Vitreous fluid collected at time of autopsy was analysed for glucose and electrolytes.	Mean age: 48 (range: 32-57)	•Males (*n* = 15)<br>•Females (*n* = 2)	•White (*n* = 2)<br>•Hispanic (*n* = 4<br>•Black (*n* = 11)	**Strengths:**<br>•First large series reporting deaths due to DKA in patients receiving atypical APDs.<br>**Limitations:**<br>•Accurate post-mortem intervals were not available for most decedents; many were discovered at home after an indeterminate period.<br>•Possible associations between APD dosage and hyperglycemia could not be evaluated.<br><br>•Missing information on APD dosage in 50% of the deaths.<br>•Medication history was not always available.

**Galler et al. (2015) [44]**<br>Inclusion:<br>•Patients with TIDM ≤ 25 years of age with a diabetes duration of > 6 months.	•Clinical characteristics and laboratory parameters were assessed including: BMI, blood pressure.<br>•Evaluated frequency of severe hypoglycemia and treatment strategy.<br>•Evaluated Frequency of episodes of DKA where the pH < 7.30, and defined according to the ISPAD.<br>•Rates of DKA and frequency of hospital admissions assessed during the previous year and calculated per 1 patient-year.<br>•Glycemic control was evaluated using the median HbA1c during the prior year. Values were measured locally and standardized according to the DCCT reference range of 4.05–6.05% (21–43 mmol/mol) using the multiple of the mean method.<br>**Exposure:**<br>•Data are shown as medians and interquartile ranges, or as means of the 95% CI.<br>•Prevalence and frequencies of complications are presented as proportions or DKA event rate (per patient-year).<br>•Kruskal-Wallis test was performed to compare median HbA1c and other variables in patients who received or did not receive treatment with an APD.<br>•Statistical Software: SAS version 9.3<br>•Poisson Model: compares the rates of DKA and frequency of hospital admissions among subjects treated with an APD.	Median age for patients with TIDM<br>•Without APD: 15.5 (IQR: 11.8, 17.6)<br>•With APD: 17.0 (IQR: 14.2, 18.3)<br>$p < 0.001$	Male/Female ratio for patients with TIDM<br>•Without APD: 52%/48%<br>•With APD:60%/40%<br>$p = 0.006$	NR	**Strengths:**<br>• First analysis that examines metabolic and glycemic control in young population < 25 years) presenting withT1DM and treated with typical and atypical APDs.<br>**Limitations:**<br>• Small sample due to the age of participants in this study. •Limited study power and associations need to be interpreted with caution.<br>•Missing information about the underlying diagnoses that required an APD prescription. The authors could not provide any interpretation on the diagnoses and their associations with metabolic or glycemic control.<br>•Cross sectional research design: descriptive and causal inference cannot be made.

	•Hierarchical random effects regression models (linear, logistic, or Poisson): diabetes center as a random effect and adjusting for age, sex, and diabetes duration were used to evaluate median HbA1c, rates of episodes of DKA and other covariates.<br>•Statistical analyses used two-sided tests. Significance: $p < 0.05$.				
**Jin et al. (2002) [45]**<br>**Inclusion:**<br>•Patients with new-onset DM associated with the use of atypical APDs.<br>**Exclusion:**<br>•Patients with a history of glucose intolerance<br>•Patients diagnosis with DM before starting the atypical APD.<br>•Patients wit-controlled DM who's condition worsened after the use of the atypical APD.	•Data was collected for: age, gender, ethnicity, family history of DM, baseline weight, weight gain, type of atypical APD and duration of exposure, number and type of adjunctive medications, blood glucose at time of diagnosis, presence of DKA.<br>•Descriptive analysis was used to evaluate the distribution of demographic characteristics.<br>•Data Analysis:<br>▪ X^2 for categorical variables.<br>▪ Student's t-test for continuous variables.	•Mean ± SD DKA cases: 37 ± 8.9	•Female: 26.3% (*n* = 19	•African American 52.6% (*n* = 19)	**Strengths:**<br>•Unique and preliminary data: the risks of new-onset DM in patients using atypical APDs<br>•Path forward for future research: the roles of ethnicity, obesity and atypical APDs.<br>**Limitations:**<br>•Small sample sizes and inconsistent reporting (missing) of demographic variables (e.g., weight gain).<br>•Reporting bias impacts the types of cases that make it to publication.<br>•Not representative of the general population of SCZ.<br>•Retrospective nature of CRs: no adequate comparison group (control) for estimation of risk.<br>•Cannot infer causality

**Koller et al. (2001; 2003; 2004) [46, 48, 49]**<br>**Koller and Doraiswamy (2002) [47]**<br>**Inclusion:**<br>Cases were identified through the MedWatch Drug Surveillance System, Medline (and psychiatry and endocrinology meeting abstracts.<br>**Exclusion:**<br>•Patients without definitive documentation were classified separately.<br>**Koller et al. (20032004):**<br>Exclusion of acidosis cases that occurred in the absence of hyperglycemia	•Database searches<br>•Assessment: Documentation of diabetes, severity of hyperglycemia, new onset of hyperglycemia, demographic features, time to onset of hyperglycemia, and effect of drug discontinuation.<br>•Newly diagnosed DM: Fasting glucose level of ≥ 126 mg/d; random glucose level ≥ 200 mg/dl; or elevated HbA1c glycohemoglobin values; and/or presence of metabolic acidosis or ketosis; the presence of frank ketoacidosis or ketosis; and/or physician institution of an antidiabetic drug.<br>• Used descriptive statistics: correlation coefficients, chi-square tests, unpaired t-tests (all two-tailed) to evaluate the relationship between variables or groups.<br>Significance: $p \leq 0.05$.<br>•Exposure: estimated by multiplying the number of prescriptions by the duration of a prescription.<br>•Reporting rates: determined from the US reports and estimates of patient exposure.	**Koller 2001**<br>Acidosis or ketosis:<br>Mean ± SD: 36±10 (range 22-57) *n* = 22<br><br>**Koller 2002**<br>47.4±12.4<br><br>**Koller 2003**<br>•Mean ± SD:<br>Risperidone : 45.8 ± 21.2 (*n* = 5)<br>Haloperidol : 48.7 ± 19.5 (n *n* = 11)<br><br>**Koller 2004**<br>For all hyperglycemic events:<br>•Mean ± SD: 35.3 ± 16.2 (range: 5–76 years) (*n* = 42)	**Koller 2001**<br>Sex Ratio (%)<br>•Male: Female 2:5 (95)<br><br>**Koller 2002**<br>Male/Female Ratio: 1:8<br><br>Addendum: 1.8:1 (*n* = 51)<br><br>**Koller 2003**<br>Acidosis: Male:Female Ratio<br>Risperidone : 0.2 (*n* = 5)<br>Haloperidol : 1.6 (*n* = 11)<br><br>**Koller 2004**<br>For all hyperglycemic events:<br>•Male: Female	**Koller 2001**<br>New-onset DM:<br>*n* = 36<br>•African descent: *n* = 21<br>•White: *n* = 8<br>•Asian: *n* = 3<br>•Caribbean: *n* = 3<br>•African-Asian: *n* = 1<br><br>**Koller 2003:**<br>NR<br><br>**Koller 2002**<br>Ethnicity available to 50 patients with high glucose levels (≥700 mg/dl):<br>•African American, *n* = 21 (42%)<br>•Caucasian, *n* = 27 (54%)<br><br>Addendum:<br>*n* = 19; 56%	**Strengths:**<br>•The FDA MedWatch system can serve as a tool for detecting post market.<br>**Limitations:**<br>•Missing information: incomplete description of cases, no control population.<br>•Biases in spontaneous adverse events databases due to underreporting, distortion in reporting.<br>•Lack of control population limits inference of causality and the ability to account for confounding factors.<br>•Insufficient information on risk factors for DM such as family history, body weight information hinders the ability to quantify risk and limits the interpretation of the findings

	•9 cases < 19 years.<br><br>Addendum:<br>•47.3 ± 18.0 (*n* = 22) overall	ratio: 1.9 (*n* = 43)	Caucasian: *n* = 10; 29%<br>African American: *n* = 3; 9 %<br>Asian: *n* = 3; 9%<br>Hispanic: *n* = 2, 6%<br><br>**Koller 2004**<br>*n* = 19<br>African American: *n* = 13<br>white: *n* = 6.<br>Asian: *n* = 3

Abbreviations: BMI, Body mass index; DCCT, Diabetes Control and Complications Trial; DKA, Diabetic ketoacidosis; DM, Diabetes Mellitus; HHS, Hyperglycemic hyperosmolar sate; ISPAD, International Society for Pediatric and Adolescent Diabetes; SD, Standard Deviation Score; T1DM, Type 1 Diabetes Mellitus; X^2, chi-square.

Symbols: > Greater than; ≥, greater than or equal to; ≤, less than or equal to.

[1]Koller et al. (2001; 2002; 2003; 2004) analyzed different hyperglycemic complications, including DKA and/or HHS. The authors did not always differentiate between all the hyperglycemic complications and demographic information is also presented for acidosis and ketosis.

[2]The analysis by Suzuki et al. (2013) [35] concluded there was no serious adverse DKA events occurred between the two groups administered: intramuscular olanzapine and intramuscular haloperidol. Therefore, it is not included in this Table.

Table S4. Risk of Bias Criteria, Ratings and Tier Classification for Analytical Studies (n = 7) and Descriptive Studies (n = 7)[1-4]

	Risk of Bias Domains and Ratings							
	Key Criteria			Other Risk of Bias criteria				
**Citation**	**Confounding Bias**	**Detection Bias 1 (Exposure Characterization)**	**Detection Bias 1 (Outcome Characterization)**	**Selection Bias (Comparison Groups)**	**Attrition/ Exclusion Bias**	**Selective Reporting bias**	**Other Biases**	**Tier**
**Analytical Studies-Cohort and Case-Control**								
Henderson et al. (2007) [36]	--	++	++	++	NR	++	-	Tier 2
Leslie & Rosenheck (2004) [37]	--	-	NR	+	++	++	-	Tier 2
Lipscombe et al. (2014) [38]	++	++	++	++	++	++	++	Tier 1
Polcwiartek et al. (2017) [39]	++	++	++	++	++	++	++	Tier 1
Ramaswamy et al. (2007) [40]	++	++	++	++	++	++	++	Tier 1
**Analytical Studies- Spontaneous Reporting**								
Liang et al. (2025) [41]	--	--	--	N/A	N/A	--	--	Tier 3
Sugarawa et al. (2023) [42]	--	--	--	N/A	N/A	--	--	Tier 3
**Descriptive Studies**								
Ely et al. (2013) [43]	--	++	++	-	++	++	++	Tier 3
Galler et al. (2015) [44]	++	-	+	++	NR	++	++	Tier 2
Jin et al. (2002) [45]	--	NR	-	--	NR	-	++	Tier 2
Koller et al. (2001) [46]	--	NR	-	--	NR	-	++	Tier 3
Koller et al. (2002) [47]	--	NR	-	--	NR	-	++	Tier 3
Koller EA et al. (2003) [48]	--	NR	-	--	++	-	++	Tier 3
Koller & Webber (2004) [49]	--	NR	-	--	++	-	++	Tier 3

[1]Adapted from Handbook for Conducting a Literature-Based Health Assessment Using OHAT Approach for Systematic Review and Evidence Integration. Office of Health Assessment and Translation (OHAT), Division of the National Toxicology Program, National Institute of Environmental Health Sciences, March 4, 2019.

[2]Risk of Bias definitions for the criteria: ++ Definitely low risk of bias; + Probably low risk of bias; --Definitely high risk of bias; - probably high risk of bias.

[3]Tiers are defined as follows: Tier 1: “definitely low” or “probably low” risk of bias for key items and “definitely low” or “probably low” risk of bias for most other applicable criteria. Tier 2: Study cannot be classified in Tier 1 or Tier 2. Tier 3” “definitely high” or “probably high” risk of bias for key items and “definitely high” or “probably high” risk of bias for most other applicable criteria.

[4]The analysis by Suzuki et al. (2013) [35] concluded there was no serious adverse DKA events occurred between the two groups administered: intramuscular olanzapine and intramuscular haloperidol. Therefore, it is not included in this Table.
